# Brewer's spent grain: Unveiling innovative applications in the food and packaging industry

**DOI:** 10.1111/1541-4337.70150

**Published:** 2025-04-02

**Authors:** Pramod Aradwad, Sharvari Raut, Ahmed Abdelfattah, Cornelia Rauh, Barbara Sturm

**Affiliations:** ^1^ Leibniz Institute for Agricultural Engineering and Bioeconomy (ATB), Max‐Eyth Allee 100 Potsdam Germany; ^2^ Indian Council of Agricultural Research, Krishi Bhavan, Dr Rajendra Prasad Rd New Delhi India; ^3^ NETZSCH Grinding & Dispersing GmbH, Sedanstraße 70 Selb Germany; ^4^ Institute of Food Biotechnology and Food Process Engineering Technische Universität, Straße des 17 Berlin Germany; ^5^ Albrecht Daniel Thaer Institute for Agricultural and Horticultural Sciences Humboldt‐Universität zu Berlin, Hinter der Reinhardtstr. 6–8 Berlin Germany

**Keywords:** brewers’ spent grain, food, nutraceutical, packaging, valorization

## Abstract

Brewer's spent grain, a byproduct of beer brewing, is often discarded as waste, leading to environmental concerns. However, the growing interest in sustainability and the circular bioeconomy has prompted research into its use in food and packaging industries. The objective of this review paper is to explore recent advancements in food applications, focusing on various aspects such as processing innovations, food properties, sensory acceptability, and safety considerations. The paper highlights the role of functional bioactive compounds of BSG in food and evaluates their pharmacological activities. Additionally, it investigates the development of sustainable food‐packaging materials derived from BSG, discussing their applications, challenges, and potential for eco‐friendly packaging solutions. The inclusion of BSG significantly impacts the food matrix during processing, which can negatively affect the physical, rheological, and textural properties and sensory acceptability. To enhance BSGs desirability as a food ingredient, various approaches have been employed, including drying, fermentation, extrusion, and modifications using enzyme treatments, dough enhancers, and texture modifiers. BSG‐derived biodegradable films and coatings demonstrate a promising potential for food‐packaging applications, offering desirable properties such as sustainability and effective performance. Key challenges for adopting BSG‐based solutions in food and packaging industries include limited consumer awareness, commercialization strategies, and the need for life cycle assessment and life cycle costing for successful integration and widespread adoption.

## 1 INTRODUCTION

The idea of a circular bioeconomy is gaining momentum as societies and industries recognize the importance of sustainable and circular practices (Baiano et al., 2023; Nyhan et al., 2023). Valorization of beer industrial byproducts is a key aspect of this approach, and it is increasing due to its positive impact on waste reduction, environmental preservation, economic development, and enhanced human welfare, especially when used as a global food supplement (Naibaho, Butula, et al., 2022a; Naibaho et al., 2023). Brewer's spent grain (BSG), a byproduct of beer brewing and spirit production (e.g., whiskey, korn), is a burgeoning resource with untapped potential. The brewing industry's global expansion has led to increased BSG generation, necessitating sustainable and efficient utilization strategies (Koirala et al., 2022; Mussatto, 2014). Significant quantities of BSG, accounting for approximately 85% of breweries waste output, are generated by both large‐scale breweries and small microbreweries alike (Sahin, Atzler, et al., 2021; Yadav et al., 2023). For every 100 L of beer brewed, approximately 20 kg of wet BSG is generated as a byproduct, with an estimated market value ranging from €35 to 50 per tonne (Assandri et al., 2021; Pabbathi et al., 2022). In 2021, global beer production was 1.89 billion hectoliters, with China leading as the largest producer at 360 million hectoliters, followed by the United States at 194 million hectoliters (Heinrich, 2023). Annually, 1.89 billion hectoliters of beer production result in the generation of 38 million tons of BSG worldwide (Hejna, 2022). According to the BarthHaas report for 2023, Germany is the leading producer of beer in Europe and 5th in the world ranking, with 87.8 million hectoliters produced by 1528 breweries, followed by Poland, Spain, and the United Kingdom (Heinrich, 2023). Europe produces approximately 10 million tons of BSG each year.

BSG is characterized by rich protein and fiber nutritional composition and generated year‐round in massive quantities, are quite inexpensive, and lack economically feasible applications (Assandri et al., 2021; Mitri et al., 2022; Naibaho, Jonuzi, et al., 2022; Petit et al., 2020; Sanches et al., 2023). Traditionally, BSG has been provided to local farmers, or it undergoes processes such as composting, drying and incineration, dumping, or anaerobic fermentation (Mitri et al., 2022; Pabbathi et al., 2022). Currently in Europe, approximately 70% of BSGs are utilized as animal feed, whereas 20% are disposed of in landfills, and 10% are used to produce biogas (Kavalopoulos et al., 2021). Improperly managed BSG waste can have harmful environmental effects due to its slow degradation under anaerobic conditions, as it is a lignocellulosic material. A major concern is the emission of methane and carbon dioxide greenhouse gases, which occur during anaerobic decomposition in open dumps or poorly managed landfills. BSG waste disposal in landfills results in greenhouse gas emissions of 513 kg CO_2_ equivalent per ton, whereas the treatment of wastewater generates 83 kg CO_2_ equivalent per ton (Fernandes et al., 2023). Water pollution is another critical concern. When BSG is disposed of improperly, rainwater can create percolate into water bodies, leading to eutrophication and harmful algal blooms.

As BSG contains high moisture content (70%–85%), preservation methods like drying and pelletizing can improve the storage and distribution, but it may also increase energy use, potentially raising GHG emissions (Ortiz et al., 2019). BSG incineration in a municipal waste facility without undergoing dewatering or drying generates emissions without yielding any energy recovery. The process is modeled using an incineration plant model for waste, where high emissions result from auxiliary materials (40%), energy (42%), and incomplete combustion (18%), leading to higher emissions compared to drying and incinerating BSG in a boiler (Scherhaufer et al., 2020). Improper disposal can also contribute to soil and air pollution. Excess nutrients and organic acids from BSG can degrade soil quality over time, whereas the decomposition of BSG releases unpleasant odors and volatile organic compounds, adversely impacting air quality (Klitkou et al., 2019). Brewing companies may face increased costs related to waste management when BSG is improperly disposed. This includes transportation and landfill fees, which could be avoided if BSG were used for its valuable components. Comprehensive waste management plans and community engagement initiatives are critical for mitigating these impacts. By implementing sustainable practices, BSG waste can be transformed into a valuable resource, reducing its environmental footprint and promoting a more sustainable future.

Due to its high moisture content (70%‐85%) and the presence of polysaccharides and proteins, BSG is highly susceptible to microbial growth and toxin development. This results in instability, a reduced shelf life, degradation of nutritional quality, and potential safety risks (Kitaw et al., 2022; Robertson, I'Anson, Brocklehurst, et al., 2010; Robertson, I'Anson, Treimo, et al., 2010). This microbial contamination is characterized by a wide range of microorganisms, with the naturally occurring microflora in fresh BSG predominantly consisted of aerobic mesophilic bacteria (2.58–6.12 log_10_ CFU/g FW), aerobic thermophilic bacteria (2.25–7 log_10_ CFU/g FW), *Pseudomonas* spp. bacteria (<2 log_10_ CFU/g FW), yeasts and molds (1.96–4.56 log_10_ CFU/g FW), microaerophilic bacteria (2.40–7.93 log_10_ CFU/g FW), and anaerobic bacteria (2.43–6.14 log_10_ CFU/g FW). The composition of these microorganisms varied across different breweries and during storage (Robertson, I'Anson, Treimo, et al., 2010). The BSG shows an increase in yeast (4.8–7.7 log_10_ CFU/g WBG)) and mold (4.8–6.6 log_10_ CFU/g WBG)) during storage at 15–25°C for 2–6 days (Kitaw et al., 2022). Similarly, in fresh BSG, the aerobic mesophilic and thermophilic bacteria were detected (2–3 log_10_ CFU/g FW) and increased to (4–8 log_10_ CFU/g FW) at 4–20°C for 0–18 days storage period (Robertson, I'Anson, Brocklehurst, et al., 2010). Moreover, molecular analysis revealed that Bacillota was the dominant bacterial phylum, whereas Ascomycota and Mucoromycota were the predominant fungal communities in raw BSG (Bianco et al., 2024). In addition, BSG feed samples collected from various dairy farms across Austria showed the presence of *Fusarium*, *Aspergillus*, and *Alternaria*, with mycotoxins such as zearalenone, T‐2, and HT‐2 were detected but at levels below European animal feed guidelines. However, *Penicillium* metabolites had the highest concentrations, suggesting potential contamination during storage (Penago et al., 2022).

The variability and composition of the BSG microbiome are influenced by factors such as raw materials, brewing techniques, temperatures, adjuncts, and storage conditions (temperature, humidity, and duration) (Bianco et al., 2024; Kitaw et al., 2022; Robertson, I'Anson, Brocklehurst, et al., 2010; Robertson, I'Anson, Treimo, et al., 2010). These factors contribute to a microflora that is highly susceptible to rapid changes after production. During the time between production and processing, which may span several hours, the bulk of hot BSG retains heat, promoting microbial growth, while limited air exposure further fosters the establishment of diverse microbial conditions. As a result, this can lead to a swift shift in the microflora, potentially causing rapid deterioration of the BSG's constituents. Therefore, a thorough understanding of the sources of variability and microbiome composition in BSG allows for prioritization based on safety risks, product quality, and intended use.

BSG contamination of fungal toxin and mycotoxin poses a significant risk to human and animal health. Samples collected from a major Argentinean brewery showed contamination with fumonisin (FB1) at levels ranging from 104 to 145 µg/kg, whereas 18% of the samples contained aflatoxin B1 (AFB1) at concentrations between 19 and 44.52 µg/kg. However, no traces of aflatoxin B2, AFG1, AFG2, or ZEA were detected (Gonzalez Pereyra et al., 2011). Similarly, samples collected from craft brewery in Belgrano, Córdoba, Argentina, showed an initial AFB1 content of 11.76 µg/kg in fresh BSG, which increased to 257 µg/kg after 7 days of storage, with a prevalence ranging from 50% to 57% (Gerbaldo et al., 2011). In another study, BSG samples collected from European Union (EU) breweries show the aflatoxin B1, B2, G1, and G2 concentrations were varied from 0.04 to 0.13, 0.08 to 0.26, 0.08 to 0.25, and 0.12 to 0.39 µg/kg, respectively (Benešová et al., 2012). Similarly, BSG samples collected from various dairy farms across Austria detected for zearalenone, T‐2, and HT‐2, though the levels were found to be below the European animal feed guidelines (Penago et al., 2022). These findings highlight the importance of addressing the contamination of BSG to ensure the safety and quality of products derived from it. Consequently, preventing the transfer of toxins through the food chain requires proper storage, handling, and monitoring of BSG toxin levels. These measures are crucial in reducing the risk of contamination and safeguarding both animal and human health.

Effective management of wet BSG postproduction involves proper storage methods (Kitaw et al., 2022; Robertson, I'Anson, Brocklehurst, et al., 2010; Wang et al., 2014) and processing techniques such as drying (Capossio et al., 2022; Domingues De Camargo et al., 2019; Pratap Singh et al., 2020; Thai et al., 2022). Other approaches, including the use of organic and inorganic acids and fermentation, have also been employed (Bianco et al., 2024; Liang & Wan, 2015; Sarkar et al., 2021; Shetty et al., 2023). These storage and processing methods are essential for maintaining BSG stability and optimizing its potential uses in food and agriculture. In‐line with the growing emphasis on sustainable practices and the circular bioeconomy, the scrutiny of waste streams has intensified, prompting researchers and industries alike to reconsider BSG as a valuable raw material.

The valorization of BSG as a food ingredient has witnessed significant advancements in recent years, driven by its potential to enhance food production, improve product quality, provide nutraceutical benefits, and contribute to sustainability. Previous reviews have addressed the composition and nutritional value of BSG (Ikram et al., 2017), its utilization in biorefineries (Agrawal et al., 2023; Colpo et al., 2022; De Paula et al., 2023; Pabbathi et al., 2022), application in food products (Lynch et al., 2016; Naibaho & Korzeniowska, 2021a; Nyhan et al., 2023; Oyedeji & Wu, 2023), in packaging application (Qazanfarzadeh et al., 2023). To date, BSG has been widely studied as an ingredient in bread, cookies, biscuits, pasta, noodles, meat analog, yogurt, fermented milk, and various other food products. Although previous reviews have largely addressed BSG‐based food products in broad terms, this review focuses on the comprehensive journey of BSG from breweries to its incorporation into diverse food products and packaging materials. Specifically, it highlights advancements in storage, drying, and processing techniques, whereas also addressing safety concerns along the way. This paper provides a step‐by‐step analysis of BSG processing, emphasizing the systematic methods required to optimize its use in food applications. The impact of incorporating BSG into these products is explored in detail, examining its effect on nutritional, physical, rheological, textural, and functional properties as well as its overall acceptability. Furthermore, the discussion extends to health benefits, offering insights into the bioactive compounds of BSG and their pharmacological activities. Beyond its role in food production, research into the application of BSG in sustainable packaging materials has emerged as a promising avenue, though it remains in its infancy. A detailed analysis examines the specific contributions of BSG components, focusing on their properties, functional characteristics, and the mechanisms by which they enhance the performance of packaging materials. In light of this, this review also explores the potential of BSG for eco‐friendly packaging, analyzing its environmental benefits, including waste reduction and its role in promoting sustainability in the packaging industry.

Through this focused approach, the review underscores the growing importance of BSG as a versatile resource, paving the way for innovative applications in food systems, packaging materials, and environmentally sustainable technologies.

## 2 COMPOSITION OF NUTRITIONAL AND BIOACTIVE COMPONENTS

BSG is an abundant lignocellulosic material containing significant amounts of cellulose, hemicellulose, and lignin, along with proteins, lipids, fat, and minerals (Mitri et al., 2022; Pabbathi et al., 2022). Its composition, however, can vary due to factors such as the type and variety of grain mixture (barley, wheat, rye, corn, etc.) and malt type, cultivation methods, brewing process, and equipment (Cadenas et al., 2021; Naibaho & Korzeniowska, 2021b; Salanță et al., 2020; Yorke et al., 2021). The detailed chemical composition of BSG is shown in Tables 1, 2, 3.

**TABLE 1:** Chemical composition of BSG.

Component	Subcomponent	Content (g/100 g)	References
Protein		12–31	Dai et al. (2022), Ikram et al. (2017), Jin et al. (2022), Santos et al. (2003), Wang et al. (2014)
Total fiber		30–70	Jin et al. (2022)
	Insoluble dietary fiber (IDF)	2.9–8.5	Jin et al. (2022)
	Hemicellulose	18–45	Lynch et al. (2016), Meneses et al. (2013)
	a. Xylan	10–21	Lynch et al. (2016), Meneses et al. (2013)
	b. Arbinian	4–9	Lynch et al. (2016), Meneses et al. (2013)
	c. Arabinoxylan	20–40	Lynch et al. (2016), Mussatto (2014), Xiros et al. (2008)
	Cellulose	10–33	Castro and Colpini (2021), Jay et al. (2008), Lynch et al. (2016), Pedro Silva et al. (2004)
	Lignin	3.5–27.8	Castro and Colpini (2021), Kitaw et al. (2022), Mussatto (2014), Pedro Silva et al. (2004), Wang et al. (2014)
	Soluble dietary fiber (IDF)	29–40	Jin et al. (2022)
	Neutral detergent fiber (NDF)	36–63	Castro and Colpini (2021), Kitaw et al. (2022), Wang et al. (2014)
	Acid detergent fiber (ADF)	13–25	Castro and Colpini (2021), Kitaw et al. (2022), Pedro Silva et al. (2004), Wang et al. (2014)
Starch		3–37	Castro and Colpini (2021), Ikram et al. (2017), Jay et al. (2008), Jin et al. (2022), Robertson, I'Anson, Treimo et al. (2010)
Lipid		3–13	Jin et al. (2022), Nazzaro et al. (2021)
Ash		2–5	Jin et al. (2022), Kitaw et al. (2022)
Total phenol content		0.5–8 mg GAE/g DW	Birsan et al. (2019), Chetrariu and Dabija (2021), Fărcaş et al. (2015), Meneses et al. (2013), Nagy and Diósi (2021), Rahman et al. (2021)
Flavonoid content		0.02–2 mg QE/g DW	Chetrariu and Dabija (2021), Fărcaş et al. (2015), Nagy and Diósi (2021), Rahman et al. (2021)
pH		3.6–6.3	Dai et al. (2022), Jin et al. (2022)

**TABLE 2:** Phenolic compounds, mineral content, and amino acids composition of BSG.

Phenolic compounds	(mg/kg DW)	Mineral content	(mg/kg DW)	Amino acid	(mg/kg DW)
Ferulic acid	1219–2017	Phosphorus	4602–6456	Histidine	21–690
*P*‐coumaric acid	125–633	Calcium	776–3600	Lysine	33–850
Catechin	1.8–29	Magnesium	1670–2564	Leucine	55–1274
Caffeic acid	9–59	Sulfur	1500–2900	Valine	63–927
Vanillic acid	11–17	Potassium	275–1617	Phenylalanines	50–881
4‐Hydroxybenzoic acid	10–15	Sodium	137–650	Isoleucines	20–565
Sinapic acid	11–25	Iron	99–190	Methonine	121–571
Syringic acid	77	Zinc	35–82	Threonine	25–519
Protocatechuic acid	3.65	Aluminum	30–82	Glutamic acid	47–839
Caffeic acid	0.28	Manganese	34–73	Aspartic acid and asparagines	13–747
		Cobalt	10–18	Arginine	33–1159
		Copper	12–50	Alanines	28–862
		Iodine	8–11	Serine	30–512
		Strontium	5–10	Tyrosines	410–1680
		Selenium	3.3–3.6	Glycines	50–349
				Proline	45–3147
Birsan et al. (2019), Jin et al. (2022), Sajib et al. (2018)		da Rosa Almeida et al. (2017), Ikram et al. (2017), Jin et al. (2022), Meneses et al. (2013), Nazzaro et al. (2021)		Jin et al. (2022), Lynch et al. (2016), Nazzaro et al. (2021)	

**TABLE 3:** Fatty acid, vitamin, and sugar composition of BSG.

Fatty acid	mg/g DW	Vitamin content	(mg/g DW)	Sugar components	% w/w Dry base
Palmitic acid	1.029–1.850	Thiamine	0.025–0.3	Maltose	0.7–8.4
Stearic acid	0.303–4.956	Riboflavin	0.025	Glucose	0.5–4.2
Oleic acid	0.041–0.072	Pyridoxine	0.009–1.1	Maltotriose	0.2–1.2
Linoleic acid	0.45–0.506	Vitamin K	0.0045	Fructose	0.3–0.7
		Niacin	0.8–1	Monosaccharides (%)	
		Cobalamin	0.25–0.55	Glucose	37
		Cholines	1800 ppm	Xylose	10.25
		Phantotenic acid	8.5 ppm	Arbinose	4.5
da Rosa Almeida et al. (2017), Tan et al. (2019)		Bianco et al. (2024), Fărcaș et al. (2022), Nagy and Diósi (2021)		Jin et al. (2022), Sajib et al. (2018)	

BSG is a significant source of dietary fiber, consisting of 40%–60% of the total dietary fiber content, with approximately 38%–58.2% classified as insoluble dietary fiber (IDF) and 1.3%–10% as soluble dietary fiber (Naibaho, Butula, et al., 2022a; Nocente et al., 2019). The primary components of IDF in BSG are cellulose (12%–33%), hemicellulose (20%–42%), and lignin (12%–22%) per 100 g of BSG, with hemicellulose notably containing 40% arabinoxylan (AX) (Gutiérrez‐Barrutia et al., 2022; Jin et al., 2022; Neylon et al., 2021b). AXs, which play a key role in various food applications such as emulsifiers, antioxidants, and prebiotics, as well as in pharmaceutical and biodegradable packaging, face certain limitations like solubility, taste, and color (Cervantes‐Ramirez et al., 2022; Lynch et al., 2016; Rojas‐Pérez et al., 2022). Furthermore, lignin (10%–28%) represents a significant component of BSG and is crucial for preserving the structural rigidity and integrity of plant cells due to its intricate composition (Naibaho & Korzeniowska, 2021b; Yadav et al., 2023).

BSG constitutes 12%–31% protein, approximately 30% of which consists of essential amino acids, with a notable abundance in lysine content (10%–15%). The variation in protein sources within BSG influences the diversity of peptides, β‐glucan levels, functional properties, and bioactivity of resulting compounds (González‐García et al., 2021; Jaeger et al., 2023; Qazanfarzadeh et al., 2023). Among these proteins, 60% is hordein (storage proteins), along with glutelin (structural proteins), albumin, and globulin, with key amino acids being leucine, proline, and glutamine (Arauzo et al., 2019; He et al., 2021; Jaeger et al., 2023). Hordein, in particular, is rich in glutamine/glutamic acid, proline, and leucine, comprising 25%, 7%, and 10%, respectively, of its weight (Shroti & Saini, 2022). The B‐hordein fraction, mainly B1‐, B2‐, and B3‐hordein, constitutes 70%–90% of total, whereas C‐hordein makes up 10%–20%, with other hordein types contributing less than 5% (Oyedeji & Wu, 2023). During mashing, BSG proteins undergo partial denaturation, leading to the formation of a complex that aggregates B‐/D‐hordein and glutelin, held together by disulfide cross‐linking bonds (Forssell et al., 2008). These gel‐like aggregates, integral to BSG protein, sit in the upper layer. These gel‐like complexes may form in the suspension and then rise to the top layer, possibly due to their density or viscosity. As a result, these interactions could influence the texture, viscosity, or clarity of the mash. Furthermore, BSG protein has limited solubility under pH 6 and reaches its highest solubility at pH 8–9 (Vieira et al., 2016). Hence, solubility impacts protein yield, quality, and amino acid composition (Cian et al., 2018). In addition to these functional properties, BSG protein demonstrates antioxidant, anti‐inflammatory, and ACE inhibitory activities, making it a superior‐quality protein with potential health benefits for diverse applications (Connolly et al., 2017; Wen et al., 2019).

The unmalted barley lipid generally ranges from 1% to 2.6%. However, during the mashing process, the lipids become more concentrated, with their levels in the BSG increasing from 3% to 13% (Niemi et al., 2012). This enrichment results in a lipid profile predominantly composed of triglyceride followed by free fatty acids comprising approximately 55%–67% and 18%–30%, respectively (Fărcaş et al., 2015; Patel et al., 2018). The specific lipid composition includes triglycerides, free fatty acids, free sterols, sterol glycosides, and sterol esters, accounting for 25,300, 6710, 910, 390, and 550 mg/kg, respectively (Del Río et al., 2013). These lipids yield compounds such as propionic and acetic acids, along with fatty acids such as palmitic, linoleic, and stearic acids, as well as tocotrienols (Parekh et al., 2017; Ribau Teixeira et al., 2020). As a result, the lipids extracted from BSG are a valuable resource for industrial applications, including nutraceutical, pharmaceutical, and cosmetic uses, as well as for other sectors such as liquid biofuel production. For instance, phytosterols found in these lipids are known to effectively reduce blood cholesterol levels, making them a practical and safe addition to foods (Zio et al., 2024). Moreover, alkylresorcinols exhibit cancer‐preventive properties by targeting cancer cells (Shestopalov et al., 2023). However, essential omega‐6 fatty acids, like linoleic acid, are essential for health and must be obtained through diet because the body cannot produce them. Linoleic acid also finds use in pharmaceuticals and cosmetics, aiding skin metabolism, enhancing vitamins A and E activity, and restoring the skin barrier (Egalini et al., 2023). Furthermore, lipids play a key role in oral drug delivery, improving bioavailability of poorly absorbed drugs through lymphatic uptake (Jeong et al., 2024).

BSG is rich in phenolic compounds like ferulic acid and *p*‐coumaric acid, which act as antioxidant and reduce chronic disease risks, including cancer, by combating intracellular oxidative stress (De Paula et al., 2023; Parekh et al., 2017). Additionally, *p*‐coumaric acid serves as a chemoprotector and precursor to beneficial compounds like xylitol and pullulan compounds, which are involved in inhibiting LDL oxidation and preventing DNA damage (Blidi et al., 2023; Thai et al., 2022). Furthermore, the presence of caffeic, sinapic, and syringic acids in BSG enhances its antioxidant, anti‐carcinogenic, and anti‐atherogenic properties (De Paula et al., 2023). Alongside these bioactive compounds, BSG is rich in minerals like calcium, magnesium, potassium, and phosphorus, as well as vitamins such as niacin, riboflavin, and thiamine. These nutritional elements contribute to various health benefits, including antioxidative, anti‐inflammatory, and immune‐modulating effects, as well as AEC enzyme inhibition (Cian et al., 2018; Connolly et al., 2019; Oyedeji & Wu, 2023; Parekh et al., 2017). Together, these properties underscore BSG's potential for health‐promoting applications in food, packaging, cosmetics, and nutraceuticals.

## 3 EFFECT OF PROCESSING TECHNIQUES ON PHYSIO‐CHEMICAL PROPERTIES OF BSG

To ensure long‐term storage and protect against unpleasant flavors and safety concerns of BSG, it is crucial to lower its moisture content under 10%, considering its initial moisture levels ranging from 70% to 85% (Capossio et al., 2022; Delfiya et al., 2022; Pratap Singh et al., 2020). There are several preservation techniques such as drying, ensilage, organic and inorganic chemicals, and other preservatives methods that had been applied to enhance the shelf life of BSG (Kitaw et al., 2022; Terefe, 2022). Among these methods, drying is extensively used in food processing and preservation. It is an energy‐intensive operation due to high thermal energy required to extract substantial amounts of water from product. More crucially, extended exposure to high temperatures is required to make shelf‐stable foodstuff, which inevitably affects food quality (Md Saleh et al., 2020; Raut et al., 2021; Sturm et al., 2020). Different drying methods such as solar, hot air, infrared, and superheated steam drying have been utilized in BSG drying mentioned in Table 4.

**TABLE 4:** Effect of drying methods on drying and nutritional components of BSG.

Drying methods	Processing conditions	Findings	References
Oven‐drying and freeze‐drying	60°C for 18 h	Oven drying BSG resulted in slight reductions in protein and fat contents compared to the wet (frozen) sample	Santos et al. (2003)
Steam drying	Steam temperature: 110–180°C Velocity: 0.25–1.08 m/s	At 145°C, increasing the steam velocity from 0.3 to 1.1 m/s lowered the drying time in half; glucan, pentosan, and protein in the dried samples remained unaffected	Tang et al. (2005)
Drum drying	Feed rate (14 and 23 kg/h), steam temperature (200 and 240°C), and steam velocity (1 and 2.5 m/s)	Energy efficiencies ranged from 60% to 76% and product moisture content between 8.8% (14 kg/h, 240°C, and 1 m/s) and 59% (23 kg/h, 200°C, and 1 m/s)	Stroem et al. (2009)
Solar dryerbiomass‐powered batch dryer	–	The biomass‐powered batch dryer required 2 h to complete drying, utilizing a biomass furnace, whereas solar drying took approximately 5.5–6 h	Mosqueda (2014)
Convective drying	Temperatures: 30, 40, and 50°C Air velocities: 0.8, 1.2, and 1.6 m/s and 70–90°C and 1.0, 1.35, and 1.7 m/s air velocity	Drying time varied between 220 and 850 min. Among all combinations, BSG (50°C and 1.6 m/s) shows less drying time (220 min)	Arranz et al. (2018)
Tray drying and pulsed fluidized bed drying (PFB)	Pulsed fluidized bed (PFB) dryer:at 900 rpm (70–90°C and 0.52, 0.72m/s air velocity)	Drying in the pulsed fluidized bed drying is faster in comparison to the tray dryer. Pulsed fluidized bed (90°C and 0.72 m/s) achieves a 90% moisture reduction that occurred twice as compared to tray dryer (90°C and 1.70 m/s)	Domingues De Camargo et al. (2019)
Vacuum microwave drying (VMD) and freeze‐drying (FD)	250 W, 180 tor, oven drier: 60–70°C, air flow rate: 0.05 m/s. FD: 1.1 mBar and −54°C)	Vacuum microwave‐dried BSG showed moderate protein functionality and the highest overall acceptability when used in baked chips	Pratap Singh et al. (2020)
Hot‐air oven and impingement drying	Temperature: 40°C for 48 h impingement: 110°C for 3.5 h	Compared to hot‐air drying, impingement drying resulted in increased protein and bioactive substances such as radical scavenging activity, total flavonoid content, and total phenolic content of BSGs and lower moisture content and water activity	Shih et al. (2020)
Kiln drying	Temperature: 50, 60, and 70°C	The drying behavior of the BSG samples was best described by the artificial neural network model, and it is also used to predict moisture values for intermediate temperatures	Bastiani et al. (2022)
Solar drying and convective drying	Temperature: 46.5°C Convective drying: 60–95°C	Drying times were 30–85 and 345–430 min for the convective drying and the solar drying, respectively. The CO_2_ emissions during convective drying varied between 294.80 and 410.73 kg/kWh for the different drying temperatures	Capossio et al. (2022)
Intermittent infrared drying and hot air drying	Temperature: 350–380°C Hot air drying: 85°C	Drying methods had showed no noteworthy impact on proximate analysis, fiber, total soluble phenolic, antioxidant capacity, and observed microbiota	Thai et al. (2022)

Limited studies have explored the impact of drying methods on nutrient preservation in BSG (Santos et al., 2003; Shih et al., 2020; Thai et al., 2022). The protein content of dried BSGs ranged from 12% to 31% (Fărcaş et al., 2015; Lynch et al., 2016; Santos et al., 2003). Studies comparing the vacuum microwave, freeze, and oven drying (65°C) methods have shown no statistically significant differences in protein concentration of dried BSG among the methods (Pratap Singh et al., 2020). Similarly, research examining superheated steam drying at different temperatures (110°C for 36 min, 145°C for 11.5 min, and 180°C for 7.5 min) and velocity (0.25–1.08 m/s) found no significant differences in protein content (Tang et al., 2005). Consistent results were observed in other studies comparing impingement drying (110°C for 3.5 h) and hot‐air drying (40°C for 48 h), showing no significant difference in protein content (Shih et al., 2020). Another study evaluated ambient (30°C), convection oven (150°C), and superheated steam drying (150°C) and similarly reported no significant differences in protein content among the methods (Cenkowski et al., 2012). Overall, these findings suggest that drying methods and temperatures generally do not affect the protein content of BSG.

However, some exceptions have been noted, such as oven drying at 65°C for 50 min, showing, 2.8%–3.5% retention of protein as compared with other two temperatures (60°C for 70 min and 70°C for 40 min) (Pratap Singh et al., 2020). In another study, infrared drying (20.69%) and hot‐air drying (21.66%) showed a statistically significant variation in protein concentration of 0.97% (Thai et al., 2022). Furthermore, protein concentration differed significantly among oven dried (60°C for 18 h) (24.2% DW), freeze‐dried (21.8% DW), and frozen BSG (26.4% DW) samples (Santos et al., 2003). In summary, although certain drying methods and conditions can have positive or negative effects on protein concentration in dried BSG, no clear trends have been established. The variability in results highlights the influence of specific drying parameters and conditions on protein retention.

No significant differences were observed in fat, ash, and carbohydrate content between BSG dried using infrared and hot‐air methods (Thai et al., 2022). Similarly, the ash content was not significant difference among oven dried, freeze‐dried, and frozen BSG samples (Santos et al., 2003). However, hot‐air‐dried BSG had higher ash content as compared to impingement‐dried ones, due to evaporation of volatile compounds and possibly some mineral losses, slightly reducing the measured ash content (Shih et al., 2020). Superheated steam drying, another alternative, caused very little change in BSG's nutrient composition, including fat and ash content (Tang et al., 2005). The fat content in BSG has been reported to range from 3% to 13% (Lynch et al., 2016). However, significant differences in fat concentration were observed among oven dried, freeze‐dried, and frozen BSG samples (Santos et al., 2003). BSG dried using the impingement method, which involves hot air under pressure and directs small jets of hot air around the food, exhibited significantly higher fat content (12.9%) compared to BSG dried with conventional hot air (9.17%). The organized arrangement of protein, water, and fat might have trapped the fat, and structural disruption in the structure caused by impingement drying as compared with hot‐air drying (Shih et al., 2020). For dietary fiber, which constitutes 45%–48% of the total composition in dried BSG, no difference was observed between the infrared‐dried and hot‐air‐dried BSG (Thai et al., 2022). Additionally, superheated steam drying of BSG at 110–180°C showed no impact on β‐glucans and pentosans, key dietary fiber components found in spent grain (Tang et al., 2005).

The drying methods have an impact on the total flavonoid content (TPC), total phenolic content (TFC), and antioxidant activity (RSA, DPPH) of a sample. Impingement drying increased the TPC, TFC, and RSA by 115%, 200%, and 58%, respectively, as compared with hot‐air drying, whereas hot‐air drying decreased the 26%, 63%, and 34%, respectively, as compared with fresh BSG (Shih et al., 2020). The high levels of TPC, RSA, and TFC are attributed to its short‐duration, high‐temperature treatment, which minimizes degradation and oxidation. The high temperature during drying may disrupt ester, ether, and acetal bonds that bind phenolic compounds, enhancing the release of bioactive compounds (Shih et al., 2020). Similarly, superheated steam drying shows higher phenol content (13 mg GAE/g) as compared with other two methods ambient and convection oven (3–4.6 mg GAE/g), due to the lack of oxygen and the higher energy intensity of steam causing more damage to the cell wall structure, making certain compounds more accessible (Cenkowski et al., 2012).

In contrary, there was an increase in percentage of total soluble phenolic and antioxidant capacity of intermittent infrared‐dried (350–380°C for 3 min, followed by 3 min off‐heat repeated for 24 min initially, and ending with 2 min of heating and 3 min off‐heat for the final 17 min) samples as compared with hot‐air drying (85°C for 2 h), but no statistical significance difference was found (Thai et al., 2022). No significant trends for total phenolic were observed for oven dried, freeze‐dried, and frozen BSG samples (Santos et al., 2003). Freeze‐drying generally maintains higher TPC and TFC values, leading to better antioxidant retention. Microwave, drying conversely, may result in some loss of phenolic and flavonoid compounds due to the rapid heating process (Pratap Singh et al., 2020).

The starch content in BSG samples decreased from 6.83% to 5.84% as the drying temperature increased from 110 to 180°C for 5–40 min and a steam velocity of 0.66 m/s (Tang et al., 2005). The elevated drying temperatures and initial moisture levels likely lead to the partial gelatinization of starch, generating amylose–lipid complexes or resistant starch. Other functional properties of dried BSG powder were affected by drying methods, with the highest water‐holding capacity observed in grains dried by oven drying at 65°C (1.52 g/g), followed by vacuum microwave (1.31 g/g) and freeze‐drying (1.18 g/g) (Santos et al., 2003). Drying methods significantly influenced color of the product. Impingement‐dried samples exhibit lower L* values than hot air‐dried ones, possibly due to the higher impingement drying temperature (110°C), resulting in a more pronounced Maillard reaction and caramelization, leading to a darker product (Shih et al., 2020). This variation in the BSG chemical composition results due to variety, harvesting, geographical location, brewing and drying temperature, method, and exposure time. Exposing the product to high temperatures during drying presents challenges in maintaining its physical, nutritional, and sensory properties. Quality evaluation at the end of processing rarely results in high‐quality food because this approach does not consider the dynamic changes in product attributes that occur during food processing (Nurkhoeriyati et al., 2022; Sturm, 2018). Non‐destructive techniques for quality evaluation are more efficient, faster, produce less waste, and are more environmentally friendly compared to traditional inspection methods. Rapid screening techniques such as spectroscopy, hyperspectral imaging, laser light backscattering, and different sensors can be utilized to assess not just the composition and quality of foods but also their safety. Quality monitoring work during drying was observed in hops (Sturm et al., 2020), carrot (Md Saleh et al., 2022; Raut et al., 2021), apple (Arefi et al., 2023), beef (Von Gersdorff et al., 2018), and potato (Amjad et al., 2018). Non‐destructive techniques like spectroscopy and hyperspectral imaging offer faster, more eco‐friendly alternatives for quality assessment.

Sticking of BSG material to the drying surface was another problem that occurred during drying, which leads to product degradation and safety risks. Drum drying at specific conditions (14 kg/h up to 0.5 m drum length) can reduce sticking 1000–1 g/m^2^ (Stroem et al., 2009), but superheated steam and freeze‐drying are safer alternatives, albeit requiring sophisticated apparatus. Apart from nutritional and other factors, optimizing energy usage and meeting energy requirements are crucial aspects in the drying process. BSG drying through rotary‐drum dryers is considered to be energy intensive because of their heat transfer mechanism, significant thermal mass, continuous operation, heat losses, and the complexity of their operation. Additionally, freeze‐drying, despite its ability to reduce volume without altering composition, is economically unfeasible due to its high energy consumption, lengthy processing times, expensive equipment, and substantial maintenance and operating costs. Oven drying is also energy intensive and requires temperatures below 60°C to avoid flavor issues. Steam velocity and temperature, and exposure time are crucial in BSG drying. In the processing of BSG, it is common for breweries to employ a two‐step drying technique. This method begins with mechanical pressing to lower the moisture content to less than 60%, followed by thermal drying to bring the moisture content below 10% (Lynch et al., 2016; Santos et al., 2003). This is not an effective method for preservation as it alters the physical characteristics and makes it more complex during drying. Other than drying, BSG cake dewatering through membrane squeezing with hot water (65°C) and vacuum application (30 min) effectively reduces moisture content from 80% to 20%, but in‐depth understanding and more research are required to validate these methods (El‐Shafey et al., 2004).

Incorporating smart drying, digital twin, non‐invasive approaches, and sensors techniques into drying can enhance not only food safety and quality control but also energy and overall performance of drying process. Future research should focus on establishing the relationship between drying parameters and nutrient content, alongside developing advanced monitoring and modeling techniques. Research on development of smart drying systems has been explored extensively in recent years (Md Saleh et al., 2020; Raut et al., 2022). The integration of a digital twin into the drying process represents a significant advancement toward smart, efficient, and quality‐controlled production method (Raut et al., 2022; Schemminger et al., 2024).

## 4 APPLICATION OF BSG IN FOOD PRODUCTS

**FIGURE 1:**
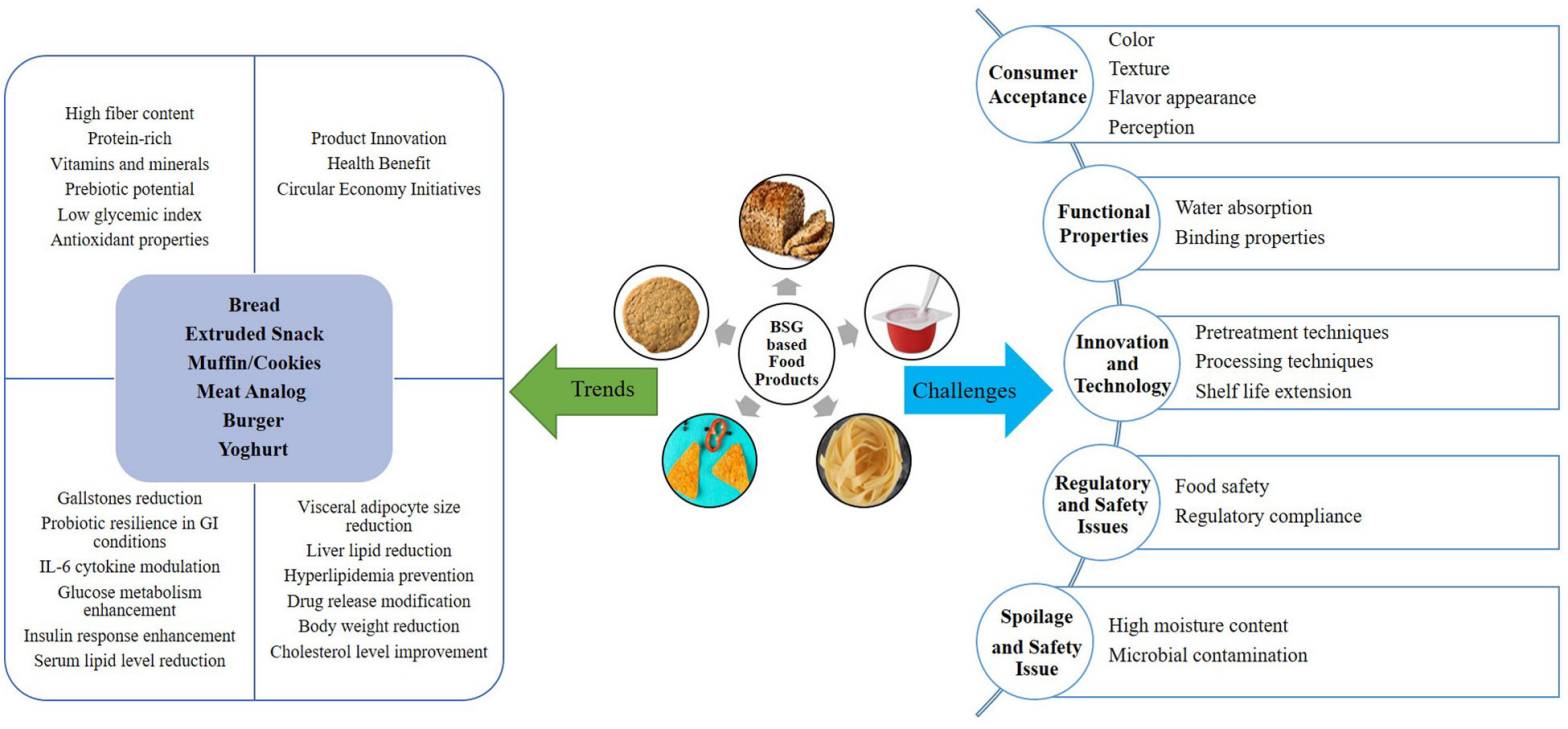
Trends and challenges in BSG‐based food products.

Bakery products, which are a vital part of a balanced daily diet, rely significantly on the use of wheat flour. Recent studies have explored the potential of nutri‐rich BSG as an ingredient in creating a variety of food products, including bread, biscuits, cookies, muffins, pasta, burger, high‐moisture meat analogs, yogurt, and beverages, aiming to harness its numerous health benefits (da Silva et al., 2024; Heredia‐Sandoval et al., 2020; Koirala et al., 2022; Madsen et al., 2021; Neylon et al., 2021b; Tan et al., 2020). The growing interest in BSG stems for its bioactive and antioxidant properties, as well as its ability to enhance bakery and pasta products when used as a partial substitute for wheat flour. This substitution includes nutraceutical ingredients, such as fiber and protein, which contribute to enhancing the nutritional profile (Figure 1) (Aprodu et al., 2017; Cappa & Alamprese, 2017; Nocente et al., 2019; Torbica et al., 2019). Table 5 provides a summary of recent food products developed using BSG and highlights the resulting changes in their nutritional profiles.

**TABLE 5:** Food product developed from BSG.

Product	Processing conditions	Findings	References
Sourdough bread	Fermentation: 75 g of BSG in 300 g water and 3% barley malt placed at 25°C and 85% RH for 24 h BSG: 15%, flour size: 250–355 µm	Sourdough fermentation in BSG breads reduces phytic acid levels, increasing the mineral bioavailability	Ktenioudaki et al. (2015)
Bread	Oven‐drying: 105°C for 78 h Milling: Flour size 400 to 600 µm BSG: 5% and 10%	BSG‐enrichment significantly boosts proteins, dietary fibers, lipids, and ash content. A 5% BSG addition enhances overall acceptance of bakery items	Amoriello et al. (2020)
Bread	Conventional oven: 60°C for 24 h Milling: flour size <250 µm Enzyme‐assisted extraction technology: hydrolysate BSG hydrolysate: 20% w/w	FBSG bread had more fiber, TPC, and antioxidant activity than control bread (CB)	Báez et al. (2021)
Sourdough bread	Fermentation: 30°C 48 h (SSF) *Aspergillus awamori* IOC‐3914	Bioprocessing breads with fermented WB and BSG resulted in a 159% and 190% increase in soluble ferulic acid, respectively	Costa et al. (2021)
Bread	Oven‐drying: 55°C for 3 h in the UF110 Plus dryer Milling: flour size below 250 µm BSG: 10% and 20%	BSG significantly increases total protein, dietary fiber, lipids, and ash content while reducing starch and bread volume compared to rye flour (RF) bread. The recommended BSG to rye flour ratio is 10%	Czubaszek et al. (2021)
Bread	Spray‐dried brewers’ spent grain (BSG): 5% and 18% fermented brewers’ spent grain (FBSG): 4% and 15%	Adding BSG and FBSG strengthens and speeds up gluten formation, reduces starch pasting capacity, and increases dough resistance and stiffness. Fermentation improves bread by increasing specific volume, reducing crumb hardness, and limiting microbial growth	Neylon et al. (2021a)
Bread	BSG: EverVita FIBRA: 4%, 5%, 11%, and 16%	EVF fiber addition enhances bread nutritional value, boosting protein by 36%, tripling fiber content, lowering pGI values by up to 25%, improving essential amino acids, protein quality, and extending microbiological shelf life	Sahin, Atzler et al. (2021)
Sourdough bread	Autoclaved (121 for 30 min) Fermentation: Mad Millie sourdough culture and incubated for 96 h at 30°C Oven drying: 60°C for 6 h FBSG: 0%–20%	During baking, a decrease in lactic and acetic acid content was noted. The most satisfactory loaves were achieved with a 10% FBSG loading	Vriesekoop et al. (2021)
Sourdough bread	Fermentation: 25°C for 24 h, *Weissella confusa* A16 in presence of sucrose	BSG breads significantly influenced the gut metabolome during in vitro digestion, promoting the production of health‐beneficial microbial metabolites	Koirala et al. (2022)
Bread	Forced‐air‐drying: 20°C for 24 h BSG: 20%–25% Milling: flour size 500 µm; <15% 500–250 µm, 35%–45% 250–125 µm, 30%–40% 125–63 µm; <15%	BSG‐enriched breads exhibited the highest phenolic and insoluble fiber percentages but had the lowest overall sensory and acceptable quality	Baiano et al. (2023)
Muffin	Drying: 110°C for 3.5 h Milling: 0.8 mm BSG: 0%–20%	BSG (15%) offered more protein, TDF, TPC, and RSA than the control muffin, maintained a brighter color than BSG (20%), and showed no significant difference in firmness or overall preference when compared to the control muffin	Shih et al. (2020)
Muffin	Enzymatically hydrolyzed BSG (carbohydrates, Biocellulase A, Bioglucanase FS2000 and Bioglucanase HAB mixed with BSG for 1 h at 50°C, incubation with Alcalase for 2 h at 50°C after which Bioprotease FV) BSG: 5%–15%	Enzymatically modified BSG muffins lowered batter viscosity and muffin hardness. Up to 10% incorporation had no adverse effect on sensory attributes	Cermeño et al. (2021)
Muffin	Mashing: 65.5°C for 1 h Drying: 49–54°C for 14 h BSG: 0%, 20%, and 30%	The appearance of the control and 20% BSG muffins was preferred over the 30% BSG muffins, whereas the 20% BSG muffins received higher flavor ratings than both the 30% BSG and control muffins	Combest and Warren (2022)
Cookies	Drying: 60°C for 12 h BSG: 20%	Adding 20% BSG to cookies improved their sensory properties, increasing protein, fiber, lipids, minerals, total phenols, and antioxidant activity. Volatile compounds such as 2‐methyl‐propanal, 3‐methyl‐butanal, and 2‐methyl‐butanal contributed to a pleasant taste and aroma appreciated by consumers	Fărcaș et al. (2021)
Cookies	Drying: 45°C for 10 h Milling: 0.4 mm mesh BSG: 0%–30%	Substitution levels significantly impacted protein content and bioactive components in cookies. Those with 20% BSG had lower hydrolysis, glycemic index, and total starch than wheat‐only cookies	Heredia‐Sandoval et al. (2020)
Cookies	Microwave treatment: 1200 W) for 2 or 6 min at 120 or 150°C Convection drying: 60°C for 12 h BSG: 15%	Microwave treatment of BSG in bakery products improves dough adhesiveness and reduces Young's modulus value of cookies by 30%, attributed to the formation of Maillard reaction products	Patrignani et al. (2021)
Pasta	Drying: 60°C for 48 h Milling: ≤500 µm sieve BSG: 3–25 g/100 g	The addition of BSG significantly decreased the average break strain of pasta, with values of 26% for raw sheets and 25% for cooked sheets, compared to 54% in both cases without BSG	Cappa and Alamprese (2017)
Pasta	Spray‐dried BSG: 2.5% and 14.96% Fermented BSG: 2 and 12.16%	Incorporating BSG and FBSG into pasta increased fiber content and predicted lower glycemic index compared to semolina pasta. It also improved pasta firmness and tensile strength compared to wholemeal pasta	Neylon et al. (2021b)
Pasta	Drying: 60°C for 72 h Milling: ≤700 µm sieve BSG: 5, 10, and 20 g/100 g	Enriched pasta saw increases in β‐glucan (up to 85%), fiber content (up to 135%), and TAC (up to 19%). Incorporating 5 g/100 g of BSG led to sensory properties comparable to durum semolina pasta, particularly regarding overall quality and firmness	Nocente et al. (2019)
Pasta	EverVita Pro (EVP—High protein): EVF: 0.8% and 9.5% EverVita FIBRA (EVF—high fibre): EVP: 1% and 12.5%	Compared to controls, EVP yielded a denser pasta structure, leading to increased hardness and tensile strength and a lower expected glycemic index	Sahin, Hardiman et al. (2021)
Pasta	Enzyme treatment: Depol 761P, incubated at 50°C for 5 h. Fermentation: *Lactobacillus plantarum* PU1 (7.5 CFU/g) 30°C for 24 h BSG and FBSG: 15%	Bioprocessing BSG enhanced protein digestibility and improved quality indices, including the essential amino acid index, biological value, protein efficiency ratio, and nutritional index, while also lowering the predicted glycemic index	Schettino et al. (2021)
Pasta	EverVita Pro (EVP—High protein): 10%–20% EverVita FIBRA (EVF—High fibre): 5%–10%	When contrasted with both 100% semolina and 100% wholegrain semolina pasta, the 15% protein‐rich and 10% fiber‐rich BSG component exhibits comparable composition, color, texture, and cooking quality	Cuomo et al. (2022)
BSG‐based bread, pasta, and chocolate milk	Convection oven: 55°C for 72 h BSG, 8.3% (w/w) in bread, 2.8% in pasta, and 0.35% in chocolate milk	BSG incorporation notably affected the sensory attributes, impacting texture and flavor. However, the fiber‐enriched bread and chocolate milk received lower overall acceptability ratings compared to their nonfiber‐enriched versions	Curutchet et al. (2021)
Bread, breadsticks, and pizza	BSG: 0%, 5%, and 10%	BSG‐enrichment notably boosted proteins, dietary fibers, lipids, and ash content in the products, resulting in significant alterations in dough rheological properties and crust and crumb color. Consumer tests identified 5% BSG enrichment as the most preferred option	Amoriello et al. (2020)
Extruded snacks and breadsticks	BSG: 0–40 g Extrusion: Speed—50 rpm, compression metering zones—150°C, and die head temperature—175°C	BSG‐enrichment substantially elevated phenolic, arabinoxylan, and TPC levels (4–7‐fold), along with DPPH (3–19‐fold) and FRAP (4–5‐fold) values in the final products. Moreover, it reduced the glycemic response from 89 to 63	Reis and Abu‐Ghannam (2014)
Snack chip	Drying: 65°C for 72 h BSG: 8%–32% Griddling: 149°C for 4 min per side Baking 176°C for 8 min	Flavor differences were not significant among the chips with varying BSG inclusion levels. BSG levels up to 40% with favorable consumer responses	Garrett et al. (2021)
Fish Burgers	BSG extract: microencapsulated (30 g/100 mL in water) at ratios of 1:2, 1:4, 1:6, and 1:8 (w/w) and subjected to spray drying at temperatures of 90, 120, and 150°C	Fish burgers formulated with a 1:2 BSG extract to capsule ratio and spray drying at 150°C exhibited exceptional chemical and sensory characteristics. These improved burgers contained 30% more polyphenols and 50% more flavonoids, displaying significantly higher antioxidant activity compared to the control sample	Spinelli et al. (2016)
Meat analog	Convective drying: 60 °C for 12 h, milling: 0.2 mm screen size BSG: 7.5%, 15%, 23.4%, and 58.5% with soy protein isolate	At 15% incorporation, BSG enhanced texturization and reduced the hardness of high moisture meat analogs (HMMAs). BSG inclusion of up to 23.4% was optimal for texturization, as higher levels reduced its effectiveness. Increased BSG levels hindered the formation of a fibrous, meat‐like structure and also darkened and browned the HMMAs but higher	da Silva et al. (2024)
Yoghurt	Convective drying Milling: <250 µm BSG: 0%–20% BSGPs were extracted using 0.5% protamex, followed by 0.1% Flavourzyme. Incubation at 50°C for 3 h (pH 8.5), followed by enzyme inactivation at 90°C	BSG reduced fermentation time, altered microstructural properties, and increased viscosity, lactic acid concentration, pH, and shear stress in yoghurt. A 10% BSG inclusion level was recommended for yoghurt preparations	Naibaho, Butula, et al. (2022a, 2022b)
Yoghurt	Liquid BSG to unsweetened soy drink mixture 20:80 and 25:75 used in the fermentation (30°C for 18 h, YFM: *Lactobacillus delbrueckii* sp. *bulgaricus* and *Streptococcus thermophiles*)	Fermenting a commercial soy drink with 20% liquid BSG yields a product closely resembling the texture and sensory characteristics of dairy yogurt with higher pH compared to non‐fermented soy drinks	Madsen et al. (2021)
Beverage	Submerged fermentation with *Bacillus subtilis* WX‐17 for 72 h at 37°C, 200 rpm	The increased levels of amino acids, total phenolic content, and antioxidant activity in the nutritious beverage	Tan et al. (2020)

Abbreviation: SSF, solid‐state fermentation.

### 4.1 Investigations of nutritional composition of BSG‐based food products

BSG supplementation has demonstrated a substantial impact on nutritional composition, blending, and viscoelastic properties, as well as fermentation behavior during preparation of bakery extruded food and beverages (Amoriello et al., 2020; Aprodu et al., 2017; Baiano et al., 2023; Costa et al., 2021). Increasing the concentration of BSG in flour blends leads to significant enhancements in protein, dietary fiber, lipids, and ash content (Czubaszek et al., 2022). Specifically, BSG at levels ranging from 5% to 40% into food formulations like bread, muffins, cookies, and pasta results in increased protein (2%–25%), ash (2.5%–65%), total dietary fiber (2‐6 fold), and fat (3%–10%) content. Simultaneously, there is a decrease in moisture content (2%–5%), starch content (10%–40%), and carbohydrate content (10%–65%) (Amoriello et al., 2020; Czubaszek et al., 2021, 2022; Fărcaș et al., 2021; Heredia‐Sandoval et al., 2020; Ktenioudaki et al., 2012; Neylon et al., 2021b; Sahin, Atzler, et al., 2021; Sahin, Hardiman, et al., 2021).

#### 4.1.1 Protein content

An increase in protein content due to BSG incorporation has been observed in bread (Czubaszek et al., 2021, 2022), muffin (Cermeño et al., 2021; Shih et al., 2020), cookies (Heredia‐Sandoval et al., 2020), and extruded snack (Neylon et al., 2021b). However, the extent of the increase varies significantly, reflecting differences in the percentage of BSG used in formulations. Protein content in BSG itself ranges widely from 12% to 31% depending on factors such as the source of barley, malting process, and brewing method (Devnani et al., 2023; Nyhan et al., 2023). EverGrain (Anheuser‐Busch InBev) has processed BSG to create two distinct ingredients: EverVita FIBRA (EVF) and PRO (EVP). EVF contains 22.8–23.4 g/100 g protein, whereas EVP offers a higher protein content of 32.2–36.8 g/100 g (Cuomo et al., 2022; Sahin, Hardiman, et al., 2021). These products also differ in fiber content (EVF: 65.8%, EVP: 46.8%) and particle size (EVF: 300–500 µm, EVP: 52–100 µm) (Cuomo et al., 2022; Sahin, Hardiman, et al., 2021). Furthermore, supplementation with EVP has been shown to significantly enhance protein levels in formulated foods, with 36% increase in bread protein content (Sahin, Atzler, et al., 2021) and 18% in pasta (Cuomo et al., 2022). Moreover, EVP improves protein quality by enhancing essential amino acid levels (Sahin, Hardiman, et al., 2021).

Studies on BSG incorporation reveal diverse effects on protein content. For instance, no significant difference in protein content was observed with up to 10% BSG in bread (Amoriello et al., 2020), 15% in muffin (Cermeño et al., 2021), 30% in bread (Stojceska & Ainsworth, 2008), and 5%–20% in pasta formulation (Nocente et al., 2019). However, significant differences were reported beyond 10% inclusion in some studies (Czubaszek et al., 2021; Heredia‐Sandoval et al., 2020; Shih et al., 2020). There is large variation in the percentage of protein level in the formulated food products. The particle size of BSG flour also plays a critical role in protein content. Cookies prepared from medium and coarse particle size (425–850 µm) exhibited lower protein content (13%–21%) compared to those made with fine flour (212–425 µm), which had a protein content of 31% (Öztürk et al., 2002). Similarly, BSG flour showed a decrease in protein content from 28.2% at a particle size of 0.21 mm to 22.7% at a particle size of 0.60 mm (Tran et al., 2020).

Processing methods, like enzymatically modified BSG (Cermeño et al., 2021) and fermented BSG (FBSG) (Báez et al., 2021; Ktenioudaki et al., 2012; Schettino et al., 2021), used in bread and pasta also did not show a significant difference of protein content compared to control. However, histidine, isoleucine, leucine, lysine, cysteine, and methionine are higher in FBSG sample than BSG and control pasta sample (Schettino et al., 2021). Similar increase of this essential amino acid compound was observed in fermentation and enzymatic hydrolysis of BSG (Chin et al., 2022). Additionally, the incorporation of 15% EVP and 10% EVF significantly improved essential amino acid levels (Cuomo et al., 2022). However, fermentation with *R. oligosporus* (37°C for 3 days) increased protein content from 25% to 34%, attributed to fungal biomass growth and enzymatic breakdown of BSG components (Chin et al., 2022). The digestibility of protein also improves with processing. FBSG was enhanced in vitro from 76% in the control sample to 88.52% in fermented sample (Schettino et al., 2021). Furthermore, BSG fermentation with *Bacillus* sp. KR‐8104 nearly doubled the alpha‐amylase content from 0.12 CU/g BSG to 0.24 CU/g in fermented samples. This increase is due to the inclusion of alpha‐amylases during fermentation, as well as the amylase production by lactic acid bacteria (LAB) during the fermentation process (Padmavathi et al., 2018). Incorporation of BSG in food formulations generally enhances protein content, and the extent of the improvement depends on factors such as the type of BSG, particle size, processing methods, and inclusion levels. Further, processing techniques like fermentation not only increase protein content but also enhance the amino acid profile and digestibility, making BSG a versatile ingredient for nutritional enrichment.

#### 4.1.2 Dietary fiber

Investigations into the addition of BSG into bread have revealed a significant increase in dietary fiber content, making these products healthier and potentially reducing the risk of chronic diseases (Amoriello et al., 2020; Czubaszek et al., 2022; Ktenioudaki et al., 2012; Shih et al., 2020). Studies have shown that incorporating 15%–20% BSG in rye or wheat bread and pasta increased the dietary fiber content by 4–5.5fold (Czubaszek et al., 2021, 2022; Schettino et al., 2021). Similarly, Fărcaş et al. (2015) found that a 5% BSG addition to wheat bread doubled the dietary fiber, whereas a 20% addition resulted in a fivefold increase. In another study, bread was supplemented with high in fiber (EVF and EVP), demonstrating 1.8–4.5 fold in fiber content (Sahin, Atzler, et al., 2021). Comparable improvements in fiber content were observed in muffins (Cermeño et al., 2021; Shih et al., 2020), bread (Czubaszek et al., 2021, 2022), and pasta formulations (Nocente et al., 2019; Cuomo et al., 2022). Furthermore, fermentation and enzymatic treatments further enhance fiber content. The addition of spray‐dried FBSG utilizing LAB increased fiber from 2 to 6.5 g/100 (Neylon et al., 2021a). Similarly, enzymatic hydrolysis of BSG‐based bread resulted in 2.5 times higher fiber compared to the control bread (Báez et al., 2021). Additionally, microbial enzymes and enzyme mixtures in FBSG may contribute to solubilizing dietary fiber, especially AX.

Pretreatment methods, such as autoclaving BSG at 95–130°C for 9–15 min, with or without applied pressure, can significantly alter the composition of soluble and IDF, making it suitable for use in functional food development (Naibaho et al., 2021). Additionally, the particle size of BSG also influences fiber levels in BSG‐enriched products. Cookies prepared with coarse particle size of BSG (425–850 µm) showed higher total dietary fiber levels compared to those with fine (<212 µm) or medium particle size BSG (212–425 µm). The fine particle size of BSG contains less dietary fiber than medium or coarse particle sizes (Öztürk et al., 2002). The percentage of fiber addition and its modification significantly influence the food texture, stability, and water‐holding capacity during processing. Additionally, fiber inclusion offers health benefits, such as lowering digestibility and promoting gut health.

#### 4.1.3 Arabinoxylan

AX is an intricate fiber that offers diverse health benefits, with its effects closely governed by its chemical composition. It plays a crucial role in food, serving not only as a source of dietary fiber with numerous health benefits but also as a functional ingredient that enhances food quality and processing. Its prebiotic, gelling, and water‐binding properties make it valuable in promoting gut health, regulating blood sugar, and enhancing food textures (Hernández‐Pinto et al., 2024; Zannini et al., 2022). Cookies with 0%–30% BSG flour alongside wheat flour exhibited AX content ranging from 0.43% to 1.08% (w/w) and arabinose/xylose ratios between 1.81 and 1.38 (Heredia‐Sandoval et al., 2020). The addition of 10%–40% BSG flour led to an increase in arabinose from 4 to 14 µg/mg, and xylose content increased by 8–15 µg/mg in the same BSG percentage range (Reis & Abu‐Ghannam, 2014). Similar amounts of AX were found in EVF‐ and EVP‐enriched spaghetti (4.5%) contained more AX than semolina pasta (2.5%), but less than wholegrain pasta (6%) (Cuomo et al., 2022). Fortifying semolina pasta with water‐extractable AX reduced its stickiness and enhanced its overall quality (Turner et al., 2008). Similarly, increasing the level of AX in cookies decreases the spread ratio and increases the hardness (Heredia‐Sandoval et al., 2020).

In bread, the inclusion of AXs at small quantity gives positive effect on specific volume and starch retrogradation; however, high doses may negatively affect texture and volume. To mitigate these drawbacks, enzyme treatment targeting AXs are commonly used, improving bread quality by extending shelf life, increasing volume, and delaying staling (Hernández‐Pinto et al., 2024; Zannini et al., 2022). Additionally, a study by Cervantes‐Ramirez et al. (2022) demonstrated that combining the extrusion process with solid‐state fermentation (SSF) using *Fusarium oxysporum* for 48 h significantly increased the release of soluble AX from brewers’ spent grain (BSG), achieving yields that were 319 times higher than those from untreated BSG. This enhanced process of AX release could be effectively incorporating into functional food development, providing the food industry to create nutritionally enriched products. However, further research is needed to fully explore the role of BSG AXs in product development.

#### 4.1.4 Ash and fat content

The incorporation of 5%–10% BSG into bakery products (bread, breadsticks, and pizza) led to a substantial increase in ash content, approximately 41% and 65%, respectively (Amoriello et al., 2020). Similarly, enzymatically modified BSG shows significant increase in ash content, ranging from 2.7% to 13.8%, compared to unmodified BSG. Additionally, increasing the BSG content from 5% to 15% further elevates ash levels (Cermeño et al., 2021). However, contrary findings were observed when 20% addition of BSG resulted in no change in ash content (Shih et al., 2020). Supporting this, Schettino et al. (2021) reported no significant distinctions in ash composition between native and FBSG. The variation in ash content in the product is primarily due to the mineral content of BSG and their ability to retain these minerals during processing, which affects the ash content in the final product.

When BSG was used as a flour replacement in cookies, replacing up to 30% of the flour showed no significant effect on fat content (Heredia‐Sandoval et al., 2020). Conversely, BSG's fat content can substitute for milk fat, thereby increasing the overall fat content to a range of 3.5%–5.1% (Naibaho, Butula, et al., 2022b). In contrast, Neylon et al. (2021b) observed a reduction in fat content in FBSG compared to its native form. Similarly, bread incorporating FBSG exhibited decreased lipid content compared to control bread (Báez et al., 2021). Furthermore, enzymatically modified BSG showed a significant decrease in lipid content (4.3%–2.5%) compared to unmodified BSG, with no changes observed when increasing BSG levels from 5% to 15% (Cermeño et al., 2021). The reduction in fat content underscores the potential of BSG as a fat replacer, offering a significant advantage in the development of healthier food formulations.

In contrast to Schettino et al. (2021), who found no significant distinctions in carbohydrate composition between native and FBSG; furthermore, the muffins made with enzymatically hydrolyzed BSG exhibit an increase in carbohydrate (9%), ash (11%), and lipid (2.5%). The enzymatic modification of BSG, facilitated by the incorporation of NaOH to enhance enzyme functionality, resulted in these increases (Cermeño et al., 2021).

#### 4.1.5 Starch

BSG contains starch, though its content is relatively low compared to other grains, typically ranging from 3% to 10% (Ktenioudaki et al., 2012), 13% (Czubaszek et al., 2021; Roberson et al. 2010), 26.64% (Castro & Colpini, 2021), and reaching as high as 37% in craft brewery BSG, as reported by Jin et al. (2022). This variation in starch content is attributed to the mashing process during brewing. The starch in BSG undergoes a unique transformation during the malting and brewing processes, which involve the partial hydrolysis. This process breaks down large starch molecules into smaller, fermentable sugars and dextrins (De Schepper & Courtin, 2024). Incorporating BSG flour into bread, bakery and extruded products has been shown to decrease the starch content of the final food products (Czubaszek et al., 2022; Ktenioudaki et al., 2012). Compared to the control cookie, the 20% additions of BSG had lower digestible starch and higher resistant starch, with BSG‐containing cookies exhibiting the lowest total starch content (Heredia‐Sandoval et al., 2020). In pasta formulations, compared to native BGS, FBSG shows an increase in total, digestible, and resistant starch, though the differences are not statistically significant (Neylon et al., 2021b). Cooking pasta led to the development of an outer layer where starch granules were highly swollen and partially broken down. This effect was more noticeable in pasta that included BSG.

In a related study, the addition of a high source of protein and fiber (EVF and PRO) in pasta formulations shows a decrease in digestible starch, resistant, and total starch. However, this modification positively influenced starch digestibility but also increased the amount of essential amino acids in the bread (Sahin, Atzler, et al., 2021; Sahin, Hardiman, et al., 2021). In cookies, a mix containing 80:20 BSG led to lower digestible starch levels and higher resistant starch compared to the control (Heredia‐Sandoval et al., 2020). In BSG, resistant starch made up 41.9% of the total starch, whereas in FBSG, it accounted for 33.9% (Neylon et al., 2021b). FBSG demonstrated a significantly higher sugar content (2.9%) compared to BSG (0.2%), which is likely attributed to the combined fermentation and hydrolysis processes utilized in FBSG production. These processes break down fibers and starches, releasing small‐chain polysaccharides and monosaccharides (Mussatto et al., 2008; Xiros & Christakopoulos, 2012).

#### 4.1.6 Antioxidant properties

Phenolic compounds in barley are primarily associated with AXs (Heredia‐Sandoval et al., 2020). Studies on   brewer's spent grain (BSG) flour have shown that both free and bound extracts exhibit higher antioxidant capacity and TPC compared to wheat flour, with these properties increasing as the concentration of BSG flour increases (Ktenioudaki et al., 2012). Furthermore, BSG‐enriched pasta, cookies, muffins, and beverages demonstrated an increase in TPC, TFC, and antioxidant potential (RSA, DPPH). Addition of 20% BSG flour to muffins significantly enhances TPC (76%) and RSA (185%) (Shih et al., 2020). Similarly, cookies with 30% BSG showed 28% increase in antioxidant activity (ATBS) and sevenfold in DPPH activity (Heredia‐Sandoval et al., 2020). A similar increase of 4 fold in TPC and 19% antioxidant activity in cookies was observed (Fărcaș et al., 2021). In pasta formulation with 20% BSG, a 13% improvement in total antioxidant capacity was achieved (Nocente et al., 2019). Similarly, 70% increase in TPC and a 23%–40% rise in antioxidant activity with 15% FBSG (Schettino et al., 2021). Extruded snacks and breadsticks with 40% BSG exhibited remarkable improvements, including a 4–7‐fold increase in TPC, 19‐fold in DPPH, and 5‐fold in FRAP (Ktenioudaki et al., 2012).

Cookies enriched with up to 30% BSG demonstrated significant increases in phenolic acids, with coumaric acid increasing from nd to 709.3 µg/g, and ferulic acid increasing from 419.2 to 3054.4 µg/g (Heredia‐Sandoval et al., 2020). Similar increases in ferulic acid from 1.32 to 2.60 mg/100 g were observed in bioprocessed bread added with FBSG (Costa et al., 2021). The predominant factor contributing to the elevated soluble ferulic acid content in breads was enzymatic bioprocessing. The enzymatic hydrolysis and fermentation of BSG in bread, the presence of *p*‐coumaric, ferulic, sinapic, and caffeic acids, along with bioactive peptides, contributed to the rise in phenol and antioxidant activity (Fărcaș et al., 2021; Schettino et al., 2021; Shih et al., 2020). Furthermore, ferulic acid exhibits a similar antioxidative effect to vitamin C, aiding in the preservation of food by preventing oxidation (Fărcaș et al., 2021).

Other treatment to BSG flour like submerged FBSG beverage containing *Bacillus subtilis* WX‐17 (Tan et al., 2020) and microwave treated BSG (Patrignani et al., 2021) exhibited an increase in DPPH and TPC content. The overall concentration of free phenolic compounds in both raw and cooked BSG and FBSG was significantly higher than control sample (Báez et al., 2021; Schettino et al., 2021). BSG treated in the microwave at 150°C for 6 min exhibits double the concentration of FRAP and DPPH than untreated BSG sample (Patrignani et al., 2021). The significant enhancement in antioxidant activity was linked to the formation of MRP (a heterogeneous group of substances) during the microwave treatment (Patrignani et al., 2021). Overall, these findings highlight the potential of BSG and its treated forms as a valuable functional ingredient to enhance phenolic content, antioxidant activity, and nutritional value in a variety of food products, paving the way for its broader application in health‐promoting and sustainable food innovations.

#### 4.1.7 Minerals and vitamins

BSG serves as a reservoir of minerals, including calcium, iron, magnesium, zinc, and potassium, and vitamins such as thiamine, niacin, pyridoxine, and cobalamin. According to Bonifácio‐Lopes et al. (2020), the mineral content in BSG may exhibit variations for phosphorus (P) content ranging from 1400 to 6000 mg/kg, calcium (Ca) content ranging from 2200 to 3515 mg/kg, magnesium (Mg) content ranging from 1900 to 2400 mg/kg, and sodium (Na) content ranging from 258.1 to 700 mg/kg. The bioaccessibility of minerals and B vitamins in BSG varies based on its composition. For instance, calcium bioaccessibility ranges from 16% to 30%, whereas vitamin B12 can reach up from 41.8% to 83% (Fărcaș et al., 2022).

Cookies with 20% BSG addition from various BSG sources showed mineral contents ranging as follows: phosphorus (2995–3035 mg/kg), calcium (1232–1271 mg/kg), magnesium (729–752 mg/kg), potassium (286–514 mg/kg), sodium (203–226 mg/kg), iron (104–131 mg/kg), and zinc (129–140 mg/kg) (Fărcaș et al., 2021). Similarly, the addition of 3%–5% BSG powder to the hybrid meat samples increases the amounts of important minerals like iron and zinc (Talens et al., 2022). Fermentation has also been shown to influence the bioavailability of nutrients in BSG. Although fermentation does not alter the overall mineral content, it may enhance the bioavailability of certain minerals (Poutanen et al., 2009). Notably, SSF of BSG at 37°C for 72 h using *R. oligosporus* enhances the pantothenic acid (vitamin B5) content by 1.84‐fold compared to unfermented BSG (Cooray & Chen, 2018). These findings highlight the nutritional potential of BSG and its fermented variants as functional ingredients in food products, contributing to improved dietary mineral and vitamin profiles.

#### 4.1.8 Antinutrients

The presence of phytic acid in BSG poses a challenge in its utilization in food products. The incorporation of BSG (10%–30%) into extruded and ready‐to‐eat snacks has been shown to increase phytic acid levels. In extruded snacks, the phytic acid content ranged from 1123 to 1561 mg/100 g with 10%–30% BSG addition (Ainsworth et al., 2007). Similarly, in ready‐to‐eat snacks, phytic acid levels increased to 1176–1610 mg/100 g, compared to the control sample, which contained 1004 mg/100 g (Stojceska et al., 2008). However, during the fermentation process of BSG, certain LAB and yeasts have been found to exhibit phytase activity. This enzymatic capability presents a promising avenue for reducing phytic acid levels. As a result of fermentation, these microorganisms can enzymatically break down phytic acid, thus alleviating its impact on nutrient bioavailability. Sourdough fermentation has been shown to effectively reduce phytic acid levels and increase mineral solubility (Water et al., 2012). The study conducted by Ktenioudaki et al. (2015) illustrates that sourdough fermentation effectively reduces phytic acid levels and enhances mineral solubility. Sourdough fermentation decreased the phytic acid content in the breads by approximately 30%, as measured in the final bread products (Ktenioudaki et al., 2015, 2012). Nonetheless, other antinutritional factors in BSG, such as tannins and oxalates, also merit further investigation to better understand their presence, roles, and overall impact on nutrient bioavailability. Addressing these challenges is essential for optimizing the use of BSG in functional food applications.

### 4.2 Investigations of rheo‐physio‐textural characterization

#### 4.2.1 Rheological properties

Incorporating BSG into bread and bakery product preparation significantly influences the entire process, starting from the initial dough preparation to the final product. This phenomenon directly impacts several key factors, as outlined below. Addition of 0%–20% BSG to wheat and rye flour results in an increase in water absorption, dough softening, and development time, with 66.2%–75%, 120–190 FU, and 0.6–6.5 min, respectively. Conversely, viscosity decrease from 1200 to 860 AU in wheat flour and from 340 to 280 AU in rye flour (Czubaszek et al., 2021, 2022). Compared to wheat flour mixed with BSG, rye flour exhibits lower water absorption, shorter development and stability times, but higher final viscosity after 30 min at 42°C. Additionally, rye dough softening reaches 175 FU, which is higher than when BSG is partially replaced with buckwheat (145 FU for 10%–20% replacement) but lower than dough made with BSG alone (185–190 FU for 10%–20% replacement). In wheat flour dough, the addition of 30% BSG leads to significant increases in development time (1.8–28.4 min), stability (12.3–50.3 min), and water absorption (54.3%–60.7%) (Heredia‐Sandoval et al., 2020). A similar trend was observed with a 20% BSG addition in bread, resulting in increased water absorption (66.2%–75%), and development time (4.1–6.5 min) but decreased stability time (6.5–3 min) (Czubaszek et al., 2022). The increase in water absorption and development time is attributed to the high fiber content of BSG in the dough. When BSG (5%–10%) is added to three different commercial wheat flours of varying strength (C1, C2, and C3), it showed varying effects on dough properties. Dough development time decreased significantly in C1 (from 17.3 to 3.5 min), C2 (from 18.5 to 3.5 min), and C3 (from 18.8 to 16.4 min). Similarly, dough stability varied, with C1 increasing from 3 to 4.2 min, C2 decreasing from 20 to 4.5 min, and C3 increasing from 2 to 12.4 min. Additionally, torque increased consistently across all samples (Amoriello et al., 2020). These results highlight that the same level of BSG addition can lead to different outcomes depending on the type of wheat flour used, emphasizing the importance of tailoring formulations to specific flour types.

The inclusion of other BSG ingredients, such as EVP and EVF (source of fiber) in bread formulations, led to a reduction in development time (4.6–2.5 min), no significant change in stability (1.27–1.50 min), and an increase in water absorption (60.4–64.3%) compared to baker's flour. In contrast, wholemeal flour and EVP, being high in fiber, exhibited longer development and stability times, as well as greater water absorption (Sahin, Hardiman, et al., 2021). BSG has been observed to bind large quantities of water and affect the density of structure that interferes with the structure formation in the production of cookies dough and bread (Ktenioudaki et al., 2012; Magabane, 2017). These observations collectively highlight the profound impact of BSG and other fiber sources on dough properties and emphasize the importance of adjusting formulations to optimize the quality and performance of bread and bakery products.

The addition of BSG and FBSG, as sources of fiber or high‐fiber ingredients, led to reductions in peak viscosity (from 1007 to 701), final viscosity (from 1327 to 643), and trough viscosity (from 607 to 321). However, breakdown viscosity was higher in FBSG (HF) (439) compared to BSG (HF) (239) (Neylon et al., 2021a). The addition of BSG and FBSG reduces the thickness of the mixture overall (less viscous), with FBSG showing greater breakdown during heating compared to BSG, likely due to differences in their composition. BSG has a high capacity to retain water and oil, which interferes with the formation of fat and protein networks (Naibaho et al., 2021; Naibaho, Butula, et al., 2022b). Viscosity of muffin batter increases with addition of BSG from 10% to 20%; this is attributed to the elevated levels of dietary fiber in BSG, serving as a thickening agent that absorbs water within the batter (Shih et al., 2020). As a consequence, water availability in the dough formation decreases, causing disruptions in network formation and gelatinized starch (Heredia‐Sandoval et al., 2020; Czubaszek et al., 2022). Likewise, adding fiber disrupts the formation of the gluten network in the dough, leading to increased swelling of starch granules and loss of substance (Cappa & Alamprese, 2017). AXs, particularly water‐extractable ones, negatively impact gluten network formation by directly interacting with gluten proteins and competing for water molecules, altering network development conditions. Unextractable AXs, renowned for their significant water‐holding capacity, have the ability to absorb water up to 10 times their own volume. The incorporation of BSG into the ingredients increases their firmness and decreases their propensity to flow (Czubaszek et al., 2022; Magabane, 2017). The incorporation of EVP and EVF, either sources of fiber or high in fiber, into pasta formulations resulted in a reduction in peak viscosity (from 697 to 391), breakdown viscosity (from 97.50 to 38.50), setback (from 853.5 to 598), and final viscosity (from 1453 to 987) with increasing levels of addition. Notably, these values in EVF‐based pasta (a fiber source) were comparable to those of semolina pasta, which exhibited the highest values among all samples, but were significantly higher than those observed in wholemeal pasta formulations (Sahin, Hardiman, et al., 2021). A similar reduction was observed in BSG and FBSG, which are rich sources of fiber and high in fiber content (Neylon et al., 2021b). The inclusion of high‐fiber ingredients (EVP and EVF) reduced starch gelatinization and enhanced the protein matrix's resistance, thereby limiting starch swelling during gelatinization. Incorporating protein and fiber (EVF and EVP) sources containing low molecular weight peptides facilitates the formation of intramolecular connections, such as hydrogen, disulfide, and ionic bonds, leading to accelerated network development. Because EVP boasts a higher protein content compared to EVF, it notably shortened the development duration more effectively. Conversely, EVF influenced the network strength to some extent. Incorporating BSG and FBSG, especially at higher inclusion levels, led to the formation of a stronger network that developed more rapidly compared to the control (Neylon et al., 2021a). The particle size of BSG also influences dough performance during mixing, affecting structure formation in bakery and extruded products. Overall, the changes in dough properties and characteristics underscore the significant role of BSG in determining the quality and processing efficiency of bakery products.

#### 4.2.2 Physical properties

Numerous studies (Amoriello et al., 2020; Baiano et al., 2023; Ktenioudaki et al., 2012; Stojceska & Ainsworth, 2008) have demonstrated that the inclusion of BSG reduces the volume of bread dough and results in bread with a dense structure. The volume reduction becomes more pronounced with a higher percentage of BSG. For instance, adding 0%–20% BSG to bread dough significantly decreases 20%–26% volume (574–355 cm^3^), and 16%–3% specific volume (3.6–2.11) (Czubaszek et al., 2021, 2022). Similar studies have found that incorporating 0%–20% BSG results in a volume reduction ranging from 20% to 60% in comparison to the control (Amoriello et al., 2020; Ktenioudaki et al., 2012; Koirala et al., 2022). The addition of extracted protein and fiber source results in a specific volume decrease ranging from 2% to 40% (Sahin, Atzler, et al., 2021). This reduction is varied with BSG hydrolysates and FBSG (Báez et al., 2021). The inclusion of BSG SD (*Lactobacillus plantarum*) boosts the bread's specific volume by 4% compared to BSG alone, accompanied by a notable increase in height of dough as compared to whole wheat dough (Waters et al., 2012).

The addition of BSG and BSG SD from 0 to 20% in wheat flour mix increases the staling rate of bread from 15.53–23.03 to 15.53–35.43, respectively (Waters et al., 2012). In contrast, breads made with FBSG using LAB exhibit a decreased staling rate, ranging from 2.10 to 0.70 (Neylon et al., 2021b). The staling rates are significantly higher in EVF (SF), EVP (SF), and EVF (HF), ranging from 2.55 to 2.70, compared to the control (1.32) and EVP (HF) (1.24), which exhibit the lowest staling rates (Sahin, Atzler, et al., 2021). The addition of medium and coarse particle sizes of 15% BSG (212–425 µm and 425–850 µm) in cookies results in a better spread ratio compared to fine particle sizes (<212 µm) (Öztürk et al., 2002). Muffins supplemented with unmodified BSG show similar height to the control, whereas 10% enzymatically hydrolyzed BSG significantly enhances 22% muffin height (3.3–4.06 cm) (Cermeño et al., 2021). Notably, the impact of BSG on bread volume varies with factors such as particle size, BSG powder, addition of FBSG, and extracted protein and fiber from BSG (Neylon et al., 2021a). AXs, prominent in BSG, contribute to the reduction in bread loaf volume by influencing gluten network construction, affecting bread quality characteristics like volume and texture (Báez et al., 2021).

The inclusion of BSG ingredients in pasta formulations affects its cooking properties, such as water absorption, optimal cooking time (OCT), and cooking losses. The addition of 5%–20% showed no change in OCT (8.5 min) as compared to control (Nocente et al., 2019). The addition of 15% BSG or FBSG results in decreased water absorption (124%–93%) and increased cooking losses (3.9%–6.9%), while maintaining a similar OCT (6–6.5 min), which is lower than that of wheat semolina pasta (9 min) (Schettino et al., 2021). However, the inclusion of BSG (2.5%–15%) and FBSG (2%–12%), whether as a source of fiber or high fiber, results in increased OCT (6–7 min) compared to wholemeal control pasta (4 min) and semolina (5.5 min) (Neylon et al., 2021b). Similarly, the addition of EVP (0.8%–9.5%) and EVF (1%–12.5%) also shows an OCT of 6.5 min for fiber‐rich formulations and 7.5 min for high‐fiber pasta, compared to semolina (5.5 min) and wholemeal flour (4 min) (Sahin, Hardiman, et al., 2021). However, 10% EVF results in a lower OCT (11 min) compared to 15% EVP and semolina pasta (∼13 min), as well as wholegrain semolina pasta (10 min) (Cuomo et al., 2022). Variation in OCT outcomes may be connected to the evolution of the gluten network and the kind of fiber. These variations can be attributed to the fiber content in BSG, which may disrupt the starch–gluten network and lead to increased starch release during cooking (Schettino et al., 2021).

Similarly, the cooking losses are influenced by the type of BSG used. For instance, FBSG results in higher cooking losses (6.9%) compared to BSG (4.8%) and wheat semolina pasta (3.9%) (Schettino et al., 2021). Cooking losses are generally higher in EVP fiber formulations (4.4%–4.6%) compared to EVF (3.5%–3.8%), with both showing overall lower losses than control samples (5.2%–6.8%) (Sahin, Hardiman, et al., 2021). However, the addition of BSG and FBSG, whether as a source of fiber or high fiber, does not show significant differences in cooking losses between the samples. The functional characteristics of pasta are greatly influenced by the amount of BSG, type of BGS (either high fiber or high protein) and FBSG used, and extrusion factors such as screw speed, type of die, and barrel temperature.

#### 4.2.3 Textural properties

Textural properties of products represent a blend of mechanical, surface, and geometrical attributes. The high dietary fiber content BSGs, mainly insoluble fiber (IDF), significantly affects the textural attributes of BSG‐enriched food products (Ačkar et al., 2018; Heredia‐Sandoval et al., 2020; Talens et al., 2022). Incorporating BSG flour enhances the hardness of various products: Cookie dough hardness increases from 44.24 to 75.15 N (Patrignani et al., 2021), 20% BSG‐enriched cookies from 37 to 42.14 N (Heredia‐Sandoval et al., 2020), 5%–20% and BSG bread from 5.33 to 11.85 N (Waters et al., 2012). The increased hardness values as compared to control are attributed due to pentosans, a fiber component in BSG, which may contribute to hardening through interactions that enhance gluten protein cross‐linking. Similarly, bread with 18% BSG exhibits hardness from 2.99 to 79.22 N (Neylon et al., 2021a) BSG fiber and protein‐based products from 4.76 to 36.36 N (Sahin, Atzler, et al., 2021). The increased hardness is attributed to the impact of the fiber content on dough development, influenced by BSGs AXs, glucan, and xylooligosaccharides, posing a challenge for dough enhancement (Aprodu et al., 2017).

However, in some cases, 3%–9% of BSG addition in sausages resulted in a decrease in hardness over time in fresh and stored samples; specifically, the hardness reduced from 12,897 to 7469 g after 7 days of storage (Mastanjević et al., 2023). In high‐moisture meat analogs, at 60% moisture content, increasing the percentage of BSG from 7.5% to 23.4% results in a decrease in hardness, although the difference is not significant. However, at 58.5% BSG, a more pronounced decrease in hardness (∼170 N) is observed, whereas the control sample exhibits the highest hardness (250 N). Similar trends were observed for chewiness. In breadsticks, increasing BSG inclusion from 0% to 35% resulted in a decrease in hardness (crispiness of the sample) (Ktenioudaki et al., 2012). Additionally, increasing the moisture content from 60% to 70% leads to a decrease in hardness across all samples, due to less interaction of diluted protein (da Silva et al., 2024). The addition of higher BSG in meat analogs disrupted the cross‐linking between SPI proteins, preventing the formation of a continuous phase essential for effective texturization.

In pasta products, the addition of BSG decreased firmness from 0.285 to 0.18 kg (Nocente et al., 2019). The diminished mechanical strength observed in BSG‐enriched pasta is ascribed to two main factors: gluten network interference and water‐soluble β‐glucan within the bran. In contrast, the firmness of pasta with a high fiber and protein content is greater than that of white flour (WM) pasta and semolina pasta. Elevated protein levels in pasta, however, are associated with increased firmness (Neylon et al., 2021b; Sahin, Hardiman, et al., 2021). High‐fiber FBSG reduced the pasta firmness compared to BSG, indicating FBSG led to this effect, possibly due to variations in gluten aggregation properties (Neylon et al., 2021b). Tensile strength, a crucial quality parameter linked to spaghetti strand elasticity, showed no significant impact when incorporating BSG‐derived EverVita a source of fiber and the control semolina. However, at a high in fiber level, a notable reduction in tensile strength was observed (Neylon et al., 2021b; Sahin, Hardiman, et al., 2021). This was attributed to changes in gluten aggregation qualities, altering pasta hardness (Neylon et al., 2021b). The tensile strength is lower in these formulations due to AXs negatively affecting gluten properties and reducing extensibility, adversely affecting pasta elasticity (Neylon et al., 2021b; Sahin, Atzler, et al., 2021; Sahin, Hardiman, et al., 2021).

Processing methods play a pivotal role in modulating the textural characteristics of BSG‐based food products. The fermentation of BSG with low inclusion levels has been shown to significantly enhance the texture of bread (softer bread) compared to un‐FBSG (Ktenioudaki et al., 2015; Waters et al., 2012). FBSG‐based pasta (Schettino et al., 2021) and fermented bread with lactic acid and added sucrose (Koirala et al., 2022) decrease the hardness compared with control. Fermentation, as demonstrated in bread and pasta, has shown consistent positive effects on texture. Moreover, altering the extrusion process and its parameters can result in variations in the hardness of the product. BSG extrudates alone exhibit high hardness, but the addition of whey protein isolate, with or without starch, decreases hardness and improves fracturability (Kirjoranta et al., 2016). Enzymatic pretreatment of BSG in muffin production notable decrease in hardness, gumminess, and chewiness, due to enhanced solubility of compounds, facilitated by protein hydrolysates and shorter chain carbohydrates (Cermeño et al., 2021). Microwave treatment at 150°C for 2 min increases the dough adhesiveness, whereas a 6‐min treatment at the same temperature decreases Young's modulus value, suggesting a texture closer to that of control products without BSG (Patrignani et al., 2021). These findings emphasize the significance of processing techniques in optimizing and modifying the textural properties of food products that contain BSG.

### 4.3 Investigations of sensory evaluation

#### 4.3.1 Color

The color of processed foods plays a crucial factor for consumer preference, making it an important consideration in product development. Fresh BSG exhibits L*, a*, and b* color values ranging from 35 to 62, 5 to 20, and 15 to 30, respectively (Fărcaș et al., 2021). These variations are influenced by the barley variety, malt type, kilning temperature, and brewing process involving Maillard reactions, caramelization, and pigment degradation (Fărcaș et al., 2021; Naibaho & Korzeniowska, 2021b). Due to its inherent brownish color, BSG is suitable for off‐white products like bread, cookies, cakes, muffins, and pasta; however, it tends to darken food products. When replacing 20% of wheat flour with BSG, the color values of white cookies changed significantly (L*: 98.84–48, a*: 1.38–10.53, b*: 11.03–21.75) (Fărcaș et al., 2021). Across all bakery products, increasing BSG levels resulted in a decrease in lightness and an increase or decrease in a* and b* parameters (Amoriello et al., 2020). In pasta, the addition of 20% BSG reduced lightness (63–38.2) and yellowness (25.4–17.9) while increasing the redness (1.25–9) (Nocente et al., 2019). Similar patterns were noted in other products like yogurt, noodles, and spaghetti (Cuomo et al., 2022; Naibaho, Butula, et al., 2022a, 2022b; Naibaho et al., 2023). These color changes are attributed to BSG's high levels of amino acids and free reducing sugars, which promote non‐enzymatic browning reactions during processing (Amoriello et al., 2020; Baiano et al., 2023; Fărcaș et al., 2021).

Post‐processing methods also play a critical role in determining the color values of BSG‐based food products. Drying, in particular, is a key factor influencing the appearance of wet BSG before it is converted into flour. Hot‐air drying at 40°C yields higher lightness compared to impingement drying at 110°C (Shih et al., 2020). Extrusion mitigates the darkening effect of BSG, as observed in changes to a* and b* color values (Thorvaldsson, 2020; Żelaziński et al., 2018). Similarly, microwave treatment prior to cookie preparation results in lower color change than adding untreated BSG (Patrignani et al., 2021). During the drying and thermal treatment process of BSG, the presence of soluble melanoidin and lignin, generated by the Maillard reaction, contributes to the darkening effect observed in BSG (Patrignani et al., 2021). Fermentation and enzymatic treatments can influence the color properties of BSG. FBSG (Depol 761P and *L. plantarum* PU1) shows increased L* and b* values and decreased a* values compared to native BSG (Schettino et al., 2021). Enzymatically hydrolyzed BSG in baked muffins alters lower color change as compared to the control (Cermeño et al., 2021).

Pasta made from EVP and EVF (as a source of fiber) derived from BSG shows higher lightness (L*) and lower redness (a*) compared to high‐fiber EVF and EVP, semolina, wholemeal, and commercially available fiber‐rich pasta. However, all pasta types exhibited similar yellowness (b*) values, except for commercial fiber‐rich pasta, which had a notably higher b* value (Sahin, Hardiman, et al., 2021). The darkening effect of BSG has significant impact on the acceptability of food products from a sensory perspective.

#### 4.3.2 Consumer acceptability

Key sensory concerns associated with the use of BSG in food products include color, texture, flavor, and overall appearance. The inclusion of BSG often results in darker color, reduced volume, increased hardness, and a denser structure (Amoriello et al., 2020; Czubaszek et al., 2021; Vriesekoop et al., 2021). However, due to changes in flavor and physical characteristics, only modest amounts (5%–20%) of BSG can be integrated into food products (Koirala et al., 2022; Sahin, Atzler, et al., 2021; Sahin, Hardiman, et al., 2021). The optimal inclusion level varies depending on the type of product and the processing methods used.

From a sensory perspective, bakery products delivered superior outcomes with 5%, whereas 10% BSG led to diminished consumer satisfaction and significant changes in aroma, color, and bitterness perceptions (Amoriello et al., 2020). In sourdough bread, sensory assessments showed that 5% FBSG resulted in the highest taste score, whereas control and 10% FBSG sourdough breads tied for second place in taste evaluation (Vriesekoop et al., 2021). Although consumers generally found 5% BSG inclusion acceptable for both modified and unmodified BSG, 10% levels introduced noticeable bitterness despite not adversely affecting other sensory attributes (Cermeño et al., 2021). In meat products, such as sausages, a 3% BSG inclusion was most preferred by consumers, whereas higher levels (4%–9%) led to less favorable evaluations (Mastanjević et al., 2023). Wheat bread incorporating more than 10% BSG exhibited post‐fermentation characteristics in aroma, taste, and texture that negatively affected its overall organoleptic quality (Czubaszek et al., 2021, 2022; Waters et al., 2012). For FBSG, both 10% and unfermented options received favorable ratings, but FBSG was perceived as less sweet.

In spaghetti, 5% and 10% BSG resulted in “good” and “sufficient” quality, but 20% exceeded acceptability thresholds, whereas micronized 20% BSG (700 µm) in pasta allowed minimal impact on processes and quality (Nocente et al., 2019). In baked snacks, exceeding 15% BSG led to unfavorable sensory ratings (Ktenioudaki et al., 2015, 2012). Similarly, incorporating 15% and 20% BSG in pasta and muffins received unfavorable sensory evaluations (Vriesekoop et al., 2021). However, in high‐moisture meat analogs, a 15% BSG incorporation level, the presence of BSG enhanced texturization and reduced the hardness (da Silva et al., 2024). When BSG inclusion exceeded 20%, significant negative effects on product attributes, including structure, texture, volume, color, and overall sensory quality. The inclusion of 30% BSG in muffins and cookies lacked flavor or had undesirable off‐notes at higher concentrations (Combest & Warren, 2022; Heredia‐Sandoval et al., 2020). Among all the products, new chips made through processes like pressing, grilling, and baking, with 40% BSG inclusion, received positive consumer feedback for overall quality (Garrett et al., 2021). Consumer tests showed that using dried BSG up to 20% matches consumer preferences like commercial alternatives. However, for optimal sensorial properties, 10%–15% BSG inclusion is considered the most acceptable.

### 4.4 Nutraceutical applications

BSG can be incorporated into the formulation of extruded snacks, enhancing the nutritional profile of the products by increasing their antioxidant and fiber content (Amoriello et al., 2020; Czubaszek et al., 2021; Reis & Abu‐Ghannam, 2014). The consumption of BSG or its derived products offers health benefits such as fecal weight, faster transit time, increased excretion of cholesterol and fats, and a reduction in the formation of gallstones (Figure 1) (Gupta et al., 2010; Ikram et al., 2017; Mussatto, 2014). BSG breads significantly affected the gut during in vitro digestion, resulting in higher production of microbial metabolites that could provide health benefits (Koirala et al., 2022). One of the key mechanisms through which BSG provides these health benefits is the enzymatic hydrolysis process using Alcalase, Flavourzyme, Corolase PP, Purazyme, and Protease P, which activates and releases the dormant small peptides that possess various bioactive properties. These bioactive peptides have shown potential in offering ACE inhibitory activity (Amorim et al., 2019; Connolly et al., 2019), anti‐inflammatory and immunomodulatory (Cian et al., 2018; Crowley et al., 2015), antihypertensive (Cermeño et al., 2019), DPP‐IV inhibitory (Baiano et al., 2023; Cermeño et al., 2019), antidiabetic effects (Connolly et al., 2017), and antioxidant (Cermeño et al., 2019; Connolly et al., 2019). The peptides hold promise for therapeutic use in managing hypertension, Type II diabetes, lowering cardiovascular risk, and addressing inflammation and disorders related to oxidative stress (Connolly et al., 2014; Crowley et al., 2015).

Moreover, BSG has demonstrated its potential as a beneficial ingredient in fermented milk, where it enhances the resilience of *Streptococcus thermophilus* TH‐4 under simulated gastrointestinal conditions, indicating its potential as a starter culture and probiotic (Battistini et al., 2021). The effect of digested BSG hydrolysate‐supplemented milk on the immune system appears to be specific to certain cells, with a more pronounced modulation of certain cytokines, such as IL‐6 (Crowley et al., 2015). Additionally, a food supplement derived from BSG extracts has been found to enhance glucose metabolism and insulin response in individuals with normal blood sugar levels, particularly those exhibiting mild insulin resistance (Ullah et al., 2022). Furthermore, microencapsulated BSG‐P has been shown to effectively reduce serum lipid levels, visceral adipocyte size, and liver lipids in rats fed a sucrose‐rich diet, indicating its potential as a natural and cost‐effective approach for preventing hyperlipidemia and associated disorders (Ferreira et al., 2022).

BSG also shows promising effects in reducing intestinal inflammation. Its liquid extract of has been found to lower the expression of inflammatory genes, including interleukin 6, interleukin 12, and MCP‐1, by 16%–53%, 43%–53%, and 57%–77%, respectively, across different concentrations (5–30 µg). It also reduces the expression of cyclooxygenase‐2, TNF‐α, and IL‐1β by 46%, 26%, and 58% at a 5 µg dose, with no further decrease at higher doses. Moreover, LPS reduced the expression of genes associated with tight junctions, such as zonula occludens‐1, claudin 1, claudin 4, and occludin. The BSG extract demonstrated potential in reducing intestinal inflammation (Darko & Kang, 2024). BSG‐aX, a fraction of BSG rich in AXs, has demonstrated potential as an excipient for modifying the release of biopharmaceutics classification system class III drugs, such as metformin hydrochloride (García‐Curiel et al., 2024). AX from brewers’ spent grain (BSG) exhibits prebiotic potential in in vitro fermentation by enhancing the beneficial growth of gut bacteria, including *Lactobacillus and Bifidobacterium* and producing short‐chain fatty acid (Lynch et al., 2021; Sajib et al., 2018).

Lignin‐rich insoluble fiber (INS) from BSG has been found to reduce body weight gain, improve cholesterol levels, and alleviate hepatic steatosis in high‐fat diet‐induced obese mice, possibly through bile acid binding or gut microbiota modulation (Raza, 2019; Raza et al., 2019). Furthermore, the cytotoxic effects of extracted protein from BSG and FBSG were evaluated on HepG2 cells using an MTT assay, with concentrations ranging from 2 to 10 mg/mL. At all concentrations, cell viability remained above 90%, suggesting that neither BSGP nor FBSGP is cytotoxic to HepG2 cells and may be suitable for food and pharmaceutical (Chin et al., 2022). Additionally, the release of small lignin‐derived molecules ilignols and catechols highlights its role in inhibiting carbohydrate fermentation, and reducing toxic components in the digestive tract, thereby lowering chronic disease risks (Aura et al., 2013). Finally, the enzymatic hydrolysis of BSG using protease showed that the anticancer screening exhibited cytotoxic effects on colon cancer cells, with IC50 values of 3.2 mg/mL for the control group and 13.91 mg/mL for Pro 1 (endoproteases). However, no notable effect was detected against brain cancer cell lines. These findings underscore the anti‐colon cancer potential of BSG extracts, highlighting their relevance for gut health (Naik et al., 2024). Similarly, BSG treated with Depol 761P and fermented with *L. plantarum* PU1 effectively protected Caco‐2 cells from oxidative stress by significantly lowering intracellular ROS levels (Schettino et al., 2021). Overall, BSG offers multiple health benefits and functional applications, making it a valuable ingredient in the development of health‐promoting food products and supplements.

## 5 ENHANCING THE QUALITIES OF BSG‐ENRICHED FOOD PRODUCTS THROUGH MODIFICATION OF BSG

Various methods, such as fermentation, enzyme treatment, dough enhancers, texture modifiers, and incorporating high‐protein and fiber from BSG, have been employed to enrich BSG‐based food products. Fermentation plays a key role in improving sensory, functional, and nutritional properties as well as shelf life (Neylon et al., 2021a; Tan et al., 2020; Vriesekoop et al., 2021). Microorganisms, through their varying abilities, significantly impact these modifications (Sahoo et al., 2023). BSG fermentation can enhance nutritional value by reducing sugar release during digestion (Neylon et al., 2021a), improving the gluten network, gas retention, and modifying gluten's physical properties through proteolytic activity (Magabane, 2017). The fermentation of BSG increases the loaf volume, improves gas‐holding capacity, and enhances texture quality by reducing firmness (Aprodu et al., 2017), while lowering free sugar content to inhibit the Maillard reaction, thus improving color (Waters et al., 2012). Additionally, fermentation enhances the solubility of dietary fiber by breaking down complex structures, producing beneficial metabolites, and making fibers more accessible to gut bacteria, which can improve overall health (Cervantes‐Ramirez et al., 2022; Vriesekoop et al., 2021). BSG acts as a prebiotic nutraceutical due to the presence of arabinoxylooligosaccharides, leading to accelerated fermentation (Amorim et al., 2019). Substituting 5%–10% BSG enhances yogurt quality, leading to optimal improvements in acidity and rheological behavior, reducing fermentation time and growth of LAB. However, inclusion at 15%–20% led to negatively impacted yogurt's flow behavior (Naibaho, Butula, et al., 2022a). Further studies by the same researcher show the addition of 10% BSG derivate in soya, and coconut‐based yoghurt shows the less fermentation time as compare to control. BSG flour and protein improved the viscosity, lactic acid production, consistency, acidity, and flow properties of soya, coconut, and milk‐based youghurt, showing they can help to maintain texture, syneresis, and form formation during 14 days of storage period (Naibaho, Butula, et al., 2022b; Naibaho, Jonuzi, et al., 2022; Naibaho et al., 2023).

In SSF with *R. oligosporus* for 72 h improves amino acids, citric acid, and vitamins (Cooray & Chen, 2018). Furthermore, the incorporation of FBSG (Depol 761P and *L. plantarum* PU1) into pasta lowers predicted glycemic index compared to un‐FBSG (Schettino et al., 2021). Fermentation also extends microbiological shelf life by 1 day, reducing released reducing sugars during in vitro starch digestion (Neylon et al., 2021a). Sourdough inclusion in bread increases acidity and total titratable acidity level (Ktenioudaki et al., 2015). Additionally, *B. subtilis* WX‐17 fermentation of BSG enhances nutritional composition, showing a 2‐fold increase in total amino acids, 1.7‐fold increase in unsaturated fatty acids, and a 5.8‐fold boost in antioxidants (Tan et al., 2019, 2020, 2021). Similarly, fermentation with *Weissella confusa* A16 produces dextran and oligosaccharides, improving wheat bread's nutritional and technological value. In another study, spontaneous fermentation (at 23°C for 72 h) followed by heat treatment (at 95°C for 60 min) and drying at 40°C resulted in a decrease in protein content (3.6%), total carbohydrate content (2%–4%), and IDF (2.9%). However, there was an increase in soluble dietary fiber (4%), phenolic content (20–25 mg GAE/g), and melanoidins (60%–80%). Additionally, these treatments led to improvements in antioxidant properties, water‐holding capacity, oil absorption, and both water and oil solubility indexes (Olvera‐Ortiz et al., 2024).

FBSG loaves with in situ dextran and maltosyl‐isomaltooligosaccharides bake better than native BSG breads (Koirala et al., 2022). In BSG fermentation, peak feruloyl esterase and xylanase activities at 1128 mU/g and 547.9 U/g, respectively, after 72 and 48 h result in diminished dough air retention capacity. However, higher xylanase, amylase, and protease activities can have the opposite effect, reducing final volume and impairing rheological attributes (Costa et al., 2021). Xylanase enzyme treatment in bread dough alters protein secondary structures, increasing the proportion of β‐turns and indicating the formation of a more effectively hydrated gluten network (Yue et al., 2022). Incorporating xylanase and dough conditioner into both sourdough and non‐sourdough‐based BSG breads enhances flour mixing and pasting properties, improves loaf‐specific volume and texture, and prolongs the onset of staleness (Ktenioudaki et al., 2015). In another application as BSG‐based emulsifier, fermented BGS with *R. oligosporus* (37°C for 3 days) followed by extraction of protein showed improved and stable emulsion properties (activity: 15–34 m^2^/g, capacity: 16%–30%, stability: 16–42 min, 7%–14%) (Chin et al., 2022).

Enzymatic treatment increases BSG‐enriched bread volume by elevating AX solubility (Ktenioudaki et al., 2012; Steinmacher et al., 2012). Xylanase‐catalyzed hydrolysis of AX into oligosaccharides enhances its prebiotic activity, with the resulting hydrolysates showing fermentation rates similar to those of commercial prebiotics (Sajib et al., 2018). Enzymes incorporated during bread making enhance textural quality. BSG hydrolysate significantly increases lipid, ash, and fiber content, with a decrease in carbohydrates and a slight decrease in protein content compared to control bread (Báez et al., 2021). The inclusion of BSG with soy protein isolates in high moisture meat analogs improves the fibrous macrostructure, texture, digestibility, and adds dietary fiber (da Silva et al., 2024). The utilization of protein and fiber sources (EVP and EVF) in bread production yields different crumb structures, decreased glycemic, and increased microbiological shelf life by up to 3 days (Sahin, Atzler, et al., 2021). The inclusion of EVP in pasta formed more robust protein network, leading to improved overall pasta quality and enhanced nutritional profile as compared to semolina (Sahin, Hardiman, et al., 2021). Similarly, pasta made with spray‐dried BSG and FBSG exhibited a superior nutritional value, featuring increased fiber content and predicted lower glycemic index compared to traditional semolina pasta (Neylon et al., 2021b). In extruded products, the addition of ingredients like pectin, egg white powder, and whey protein enhances the finished BSG product's quality (Cappa & Alamprese, 2017; Kirjoranta et al., 2016).

## 6 APPLICATION OF BSG IN DEVELOPMENT OF PACKAGING MATERIAL

The biodegradable packaging material developed using polysaccharides, proteins, and lipids extracted from different agro‐industrial waste presents a promising solution to the sustainability challenges faced by the packaging sector (Assad et al., 2020). The use of these extracted compounds in packaging development holds promise for enhancing the barrier, thermal, mechanical, optical, and antibacterial properties of biodegradable packaging. In particular, proteins have gained significant attention among biopolymer materials due to their ability to form networks, impart plasticity and elasticity, act as effective oxygen barriers, offer mechanical capabilities, affordability, edibility, and rapid biodegradability (Assad et al., 2020; Yashwant et al., 2023).

BSG is protein‐rich, featuring a variety of amino acids. The diverse protein structures foster interactions that contribute to its superior mechanical properties. Researchers explored the potential of BSG protein in development of packaging films and coatings (Table 6). In a study by Lee et al. (2015), a composite film combining BSG proteins and chitosan was investigated, noting that increasing chitosan concentration (0%–100%) resulted in changes in properties. The film exhibited decreased water vapor permeability (WVP), elongation at break, and water solubility, while demonstrating increased tensile strength, color intensity, antioxidant activity, and antibacterial effectiveness. The formation of intermolecular hydrogen bonds between BSG protein and chitosan improved tensile strength (Lee et al., 2015). Similarly, Proaño et al. (2020) found that the formulations containing 0.10 and 0.15 g of polyethylene glycol (PEG) per gram of BSG‐polycarbonate produced films with well‐balanced combination of water‐barrier properties, antioxidant capacity, and mechanical strength in the resulting films. PEG increased the α‐helix structure up to addition of 0.10 g, improving the mechanical properties and antioxidant activity, although no antibacterial effects against *Penicillium corylophylum*, *Bacillus cereus*, or *Salmonella* Newport were observed. The remarkable antioxidant activity of BSG protein was attributed to the presence of oligopeptides and free amino acids. These emphasize the potential of BSG proteins in developing composite films with desirable properties for various applications, including packaging. Shroti and Saini (2022) investigated the BSG protein‐based films prepared with different concentrations of protein (4%–10%) at varying pH levels (11–13); the developed films exhibited a range of colors, from opaque to light reddish‐brown. Increasing the protein concentrations in the films led to improvements in mechanical characteristics, moisture content, and water activity. However, an increase in pH resulted in enhanced solubility but reduced water activity, swelling capacity, and WVP. The BSG protein films also exhibited UV‐blocking characteristics, which were attributed to the ability of their aromatic amino acid residues to absorb UV light (Limpan et al., 2010; Shroti & Saini, 2022). Despite their slightly reddish‐brown color and poor transparency, the characteristics of the BSG protein films were promising.

**TABLE 6:** Use of BSG in packaging film/coating.

BSG material and type of film	Composition	Properties			Findings	References
Composite films	BSG protein (BGP): 3% Chitosan (Ch): 2% Ratios: 100BGP:0Ch, 70:30, 50:50, 30:70, and 0BGP:100Ch), 40% (w/w, BGP basis), glycerol: 40% (w/w)		Composite films	Control (chitosan)	• The optimal composite film of BGP:Ch in a 50:50 ratio, exhibiting good TS, EAB, and WVP values of 23.71 MPa, 36.73%, and 2.82 g m/(m^2^ s Pa), respectively. Additionally, it demonstrated antimicrobial and antioxidant activities, with values ranging from 15.54 to 16.70 mm and 5.44%, respectively • The addition of chitosan to the BGP composite films improved their antibacterial and antioxidant capabilities	Lee et al. (2015)
		TS (MPa)	∼4.32–26.20	41.95		
		EL (%)	∼28.54–54.55	7.16		
		WVP (×10^−9^ g m/(m^2^ s Pa))	∼2.72–2.93	7.16		
		WS (%)	∼21.54–37.56	10.59		
		Δ*E*	∼7.62–20.87	49.78		
		WI	46.38 –63.63	95.49		
		Opacity	8.32–24.82	1.22		
		Inhibition zone (mm)				
		*Staphylococcus aureus*	0–19.09	25.57		
		*Listeria Monocytogenes*	0–18.63	21.22		
		*Escherichia coli* O157:H7	0–17.15	19.17		
		*Salmonella typhimurium*	0 –17.22	18.18		
BSG proteins basedbiodegradableactive film	Protein concentrate: 75 g/L pH: 2 Polyethylene glycol (PEG): 0.05–0.25 g/g		Active film	Control: without PEG	• Due to the protein's strong interaction with water, increasing PEG concentration from 0 to 25 wt.% increased film opacity, solubility, and WVP • The formulation of 0.10 and 0.15 g PEG per gram of BSG‐PC demonstrated an optimal balance between the mechanical properties, water‐barrier characteristics, and antioxidant capacity of these films	Proaño et al. (2020)
		MC (g/kg)	95.4–148.1	95.6		
		WS (g/kg)	704–897.8	ND		
		WVP (g H_2_O/(Pa s m)) × 10^−10^	ND–11.6	ND		
		Thickness (µm)	83.10–106.84	95.60		
		Δ*E*	61.44–66.45	62.47		
		Opacity (AU/mm)	9.83–15.63	11.52		
		TS (MPa)	0.3–1.3	–		
		EL (%)	23–40	–		
		ABTS•+ radical (µm Trolox/g protein)	1100–1300	∼900		
		Reducing power (mg ascorbic acid/g protein)	∼600–700	∼600		
		β‐Carotene bleaching inhibition (mg BHT/g protein)	∼2.8–3.5	∼2.8		
Protein‐edible film Monolayer	Dried BSG protein content: 76% w/w db Protein concentration: 4%, 6%, 8%, and 10% pH levels: 11, 12, and 13 and glycerol: 30 wt.%)	pH solubility (%)	49.08–65.38	–	• By elevating the pH level and protein concentration, the tensile and puncture strengths improved • The characteristics of film were low transparency and reddish‐brown color • Increasing pH decreases the WVP, whereas increasing protein concentration increases the WVP	Shroti and Saini (2022)
		MC (%)	17.79–29.69	–		
		Swelling capacity (%)	415.91–728.15	–		
		Aw	0.364–0.469	–		
		Transparency (A600/x)	5.56–9.74	–		
		TS (MPa)	∼0.75–1.4	–		
		EAB (%)	∼2–55	–		
		PS (MPa)	∼0.14–0.42	–		
		WVTR (g/m^2^ h)	29.36–40.84	–		
		WVP (g/m s Pa) × 10^−10^	3.03–4.58	–		
		Permeance (g/m^2^ s Pa) × 10^−6^	1.44–2.18	–		
		Δ*E*	64.20–72.17	–		
Arabinoxylans (AX) Nanocomposite films	AX: 2% w/v Nano fibrillated cellulose (NFC): 5–75% mass fraction, (AX andNFC, viz. 95:5 to 25:75 wt.%), ferulic acid (FA): 75 mg/g, and feruloylated‐arabinoxylooligosaccharides (FAXOS): 75 mg/g		Nanocomposite films	Control: arabinoxylans	• The AX‐NFC50 film offers good mechanical characteristics, including temperature stability up to 230°C and a Young's modulus of 7.5 GPa • The AX50:NFC50 with FA and FAXOS film exhibits a notable antioxidant activity of up to 90%, strong UV–vis barrier properties, antifungal effects against *Candida albicans*, and antibacterial activity against *Staphylococcus aureus* and *Escherichia coli*	Moreirinha et al. (2020)
		TS (MPa)	10.1–116.8	81.7		
		EAB (%)	1.29–3.16	3.43		
		YM (GPa)	4.23–7.53	4.31		
		Color values (L*, a* b*)	45.11–52.63, −0.45 to 0.84, −2.08 to 11.26	44.19, 0.04, 6.11		
		Total weight loss (%)	25.28–35.28	13–30		
		Antimicrobial activity (log (CFU/mL))				
		*S. aureus*		∼4.5–9		
		*E. coli*		∼7–9		
		*C. albicans*		∼4–5		
		Antioxidant activity (%)		64–90		
BSG‐AX basedthermoplastic films	AX: 5% w/v	Total weight loss (%)	25.28–35.28		• Plasticized films manufactured with BSG‐AX exhibit resistance to a broad temperature range • Total coliform growth was not seen, and only a little amount of aerobic mesophylls (10^2^ CFU/g) was found in BSG‐AX film	Jaguey‐Hernández et al. (2022)
		Enthalpy, Δ*G* (kJ/mol)	174.13–184.68			
		Gibbs free energy, Δ*H* (kJ/mol	34.29–55.35			
Carboxyl methylcellulose (CMC)composite	Protein: 13.2–52.8 mg PhCs: 5–20 mg Gly: 10% v/v			Control: CMC without protein and phenolic compounds	• The protein and phenolic in the CMC control films reduced the tensile strength modulus of elasticity and elongation at break • The appearance of composite film‐coated strawberries was superior to that of uncoated films after 5 days of storage at room temperature.	Oztuna Taner et al. (2023)
		YM (MPa)	8.32–14.08	16.31		
		TS (MPa)	1.19–1.30	5.36		
		EAB (%)	8.80–16	32.84		
		Antioxidant activity (DPPH assay) (%)	∼26%–29%			
BSG fibers Nanocomposite films	BSG: 5 or 10 wt.% Cassava based thermoplastic starch: 8% w/v Lemongrass oil (1:1), glycerol: 20 wt.%, and cocoa butter: 30 wt.%			Control: thermoplastic starch	• An increase in fiber concentration (5%–10%) led to a reduction in the tensile strength, lightness of film, hydrogen bond formation, WVTR, WVP, and elastic modulus while increasing thickness and elongation at break • Films on BSG fibers containing upto 5% BSG demonstrated an acceptable surface and can be used as edible packaging material	Mendes et al. (2021)
		Thickness (µm)	142.5–230.4	50.2		
		YM (MPa)	1.2–13	8		
		TS (MPa)	3.6–12	21		
		EAB (%)	2.8–9.4	2.4		
		WVTR (g/h/m^2^)	30–45	62		
		WVP (g mm/(kPa h m^2^))	2.4–2.9	4		
		Δ*E*	14.9–30.9	4.82		
BSG fibers Biodegradable films	BSG: 0–5 g (35, 60, and 100 mesh) Corn starch:10 g Glycerol: 10 g	Aspect ratio L/D	1.53–2.01	–	• Incorporating BSG into pure TPS can lead to a modest improvement in its tensile strength and thermal stability. This makes BSG a potential active filler because it is compatible with the polymer structure and makes the polymer crystallize more • BSG (2.5 wt.% 100 mesh) can reinforce composites, improving tensile strength and thermal stability	Castanho et al. (2022)
		Surface area‐to‐volume ratio SA/V (mm^−1^)	15–43	–		
		MC (%)	0.44–0.62			
		Thickness (mm	0.25–0.38	0.31		
		TS (MPa)	0.73–0.94	0.83		
		*E*_a_ (%)	12.13–24.10	122.35		
		*T*_m_ (°C)	139–151	135		
		Δ*H*_m_ (J/g)	38–129	46		
		Thermal residue (%)	7.1–8.1	3.2		
Dried BSG Disposable trays	BSG: 20–80 g Potato starch: 16.7–76.7 g Glycerol: 3.3 g Gelatin: 1 Chitosan: 3 g	Flexural strength (MPa)	∼2.2–4.3	–	The highest flexural strength was shown by trays made with 60% BSG, as well as by adding chitosan and glyoxal, with respective values of 3.75 MPa and 0.52 MPa before and after contact with water	Ferreira et al. (2019)
		Flexural strain (%)	∼1.1–2	–		
		Flexural modulus (MPa)	∼1.1–2.1	–		
		Water absorption (%)	12–16	–		
BSG powderfortified bio‑based edible bowls	BSG: 0, 1%, 2%, 5%, and 10% Ragi and refined flour: 50:50 ratio Jaggery:30 g Xanthan gum (1 g)	Water absorption (%)	3.31–11	–	BSG (10%) added bowl sample has low water absorption and oil absorption and higher antioxidant and hardness properties	Nehra et al. (2022)
		Oil absorption (%)	5.83–18	–		
		Antioxidant activity (%)	65–85	–		
		Hardness (kgf)	10–20	–		
Biodegradable composites	Cassava starch: 3% w/w BSG: 3% w/w Glycerol: 20% w/w	Thickness (mm)	0.060	0.070–0.085	• The addition of BSG fiber enhances tensile strength by 80% and increases Young's modulus by 50% Bleaching and mercerization treatments of BSG improved the water solubility and WVP in fiber‐reinforced composites	Ramos et al. (2024)
		TS (MPa)	10.23	3.98–18.5		
		EAB (%)	13.71	4.4–83.8		
		YM (MPa)	175.01	124.9–292.1		
		Water solubility (%)	32	19–63		
		WVP (10^−11^ g/(m s Pa))	2.45	1.2–7.45		
Biodegradable active packaging	Cassava starch: 3% w/w BSG extract: 5%–10% w/w Glycerol: 20% w/w	Thickness (µm)	49.5	49.5–87.5	• Increasing the % of BSG extract in active film shows increasing resulted in greater thickness and density, with no significant changes in opacity, water solubility, tensile strength, or Young's modulus • BSGE had no impact on the microstructure or mechanical properties of the films, maintaining consistent resistance even at higher concentrations	Vieira et al. (2024)
		Density (g/cm^3^)	0.0039	0.0052–0.079		
		Opacity (%)	29	20.6–32.9		
		Water solubility (%)	47.64	48.78–54.1		
		WVP (g/(m s Pa)) × 10^−13^	9.78	5.53–8.11		
		TS (MPa)	5.33	5.08–5.73		
		EAB (%)	262.1	155–232.4		
		YM (MPa)	130.3	154.6–182.2		

In addition to proteins, carbohydrates, such as cellulose, AXs, glucan, and starch, serve as excellent biomaterials for food packaging due to their biodegradability, intricate network structure, high water resistance, and remarkable mechanical properties (He et al., 2021). AXs and carboxymethylcellulose (CMC) from BSG have been explored in composite films (Jaguey‐Hernández et al., 2022; Moreirinha et al., 2020; Oztuna Taner et al., 2023). The incorporation of AXs extracted from BSG into methyl hydroxyethyl cellulose‐based films enhanced thermal stability, hydrophobicity, WVP, and antioxidant activity (50% DPPH inhibition), while preserving transparency and slightly reducing mechanical properties. These functional films demonstrate significant potential for use in active food packaging to extend shelf life (Rojas‐Lema et al., 2024). Additionally, BSG‐derived feruloylated‐arabinoxylooligosaccharides with various cellulose nanofiber (CNF) concentrations displayed a translucent and thermally stable film with good mechanical properties and a pale‐yellow hue (Moreirinha et al., 2020). This stronger interaction between AXs and NFC resulted in increased surface adhesion, yielding stiffer materials and enhancing mechanical performance. AX film showed 0%–37% and 37%–57% transmittance in the visible and ultraviolet, respectively. It also improved antioxidant activity, antibacterial (*Staphylococcus aureus* and *Escherichia coli*), and antifungal activity (*Candida albicans)* (Moreirinha et al., 2020). In a separate study conducted by Jaguey‐Hernández et al. (2022), thermoplastic films based on BSG AX resulted in homogeneous, clear films with a warm amber hue and excellent thermal stability across a pH span of 3–10. The addition of plasticizer and defoamer agents increased the activation energy (*E*_a_) of the films. The film derived from CMC exhibited improved mechanical properties and increased antioxidant activity. The incorporation of BSG protein and phenolic constituents notably impacted mechanical attributes, affecting the elongation at break and modulus of elasticity. This was attributed to the reduced molecular mobility of CMC polymeric chains, resulting in a more rigid structure. However, the addition of carboxymethyl groups to the cellulose chain, along with the presence of negatively charged carboxymethyl groups, resulted in a reduction in the thermal stability of CMC (Oztuna Taner et al., 2023).

In another exploration by Mendes et al. (2021), BSG fibers were incorporated into a quaternary nanocomposite film with cassava starch, cocoa butter, and lemongrass essential oil, and 5%–10% fibers in the film reduced the lightness, tensile strength, WVP, hydrogen bond formation, water vapor transmission rate, and elastic modulus, while increasing thickness and elongation at break. The inclusion of BSG fibers, acting as a dispersed phase, restricted the movement of biopolymer chains, consequently leading to reduced permeability of water vapor in composite films. After a 45‐day biodegradability test (dried at 50°C in a vacuum oven), the material began to degrade, displaying toning down, disintegration, and deposition of organic debris on the surface. In a related investigation by Castanho et al. (2022), BSG fiber‐based film compositions exhibited better mechanical properties, emphasizing the influence of the interplay between phases and fiber size on the mechanical performance of the films. In contrast to pure thermoplastic starch, the presence of BSG fibers facilitated a prolonged retrogradation process, resulting in elevated melting temperatures and heightened crystallinity within the composites. These composites maintained their structural integrity at temperatures below 130°C (Castanho et al., 2022).

Furthermore, incorporating pretreated BSG, subjected to mercerization (10% w/v NaOH) and bleaching (10% w/v NaOH and 12% w/v H_2_O_2_), into starch‐based composites improves tensile strength, WVP, Young's modulus, and water solubility (Ramos et al., 2024). While incorporating BSG extract into starch‐based films had no significant impact on their mechanical performance or barrier properties, it resulted in a rough and heterogeneous surface texture (Ludka et al., 2024; Vieira et al., 2024).

In another packaging application, Ferreira et al. (2019) investigated BSG‐based food‐packaging trays made from BSG, incorporating chitosan and glyoxal, and demonstrated increased flexural strength, both pre‐ and post‐water exposure, approaching the performance of expanded polystyrene trays. In a separate study by Nehra et al. (2022) on BSG‐based edible bowls fortified with ragi, refined flour, xanthan gum, and jaggery, 10% BSG addition exhibited low water and oil absorption, and superior antioxidant and hardness properties. This suggests that BSG could serve as a cost‐effective solution for creating biodegradable packaging materials.

BSG‐based packaging materials were evaluated for primary packaging of food, starting with chocolates in a study by Mendes et al. (2021). Dark and white chocolates were wrapped in BSG‐based nanocomposite films, covered in aluminum foil, and stored at room temperature for 7 days. Sensory evaluation indicated that films containing 5% BSG fiber were more accepted for both types of chocolates compare to 10% BSG due to active component migration from the films. In another study, Oztuna Taner et al. (2023) coated strawberries by dipping them into a solution of BSG protein (13.2–52.8 mg) and PhCs (5–20 mg) for 2 min. After air drying at room temperature and storing for 5 days at 18°C, the coated strawberry samples displayed superior appearance properties compared to uncoated ones, attributed to the protective qualities of the films. The coated samples showed lower weight loss and maintained better levels of solid soluble (Brix), acidity, pH, and anthocyanin values, indicating the composite film's effective 5‐day protection for coated strawberries. Furthermore, BSG‐derived CNF cryogels, combined with PEG, were used in composite films for frozen meat packaging. These composites exhibited excellent shape stability, highest enthalpy values, and superior mechanical properties. They effectively prevented rapid temperature fluctuations and reached the meat temperature below 0°C for four times longer than the control package, with no leakage during phase transition. Additionally, the composite positively impacted drip loss and color stability during temperature changes (Heidari et al., 2025).

**FIGURE 2:**
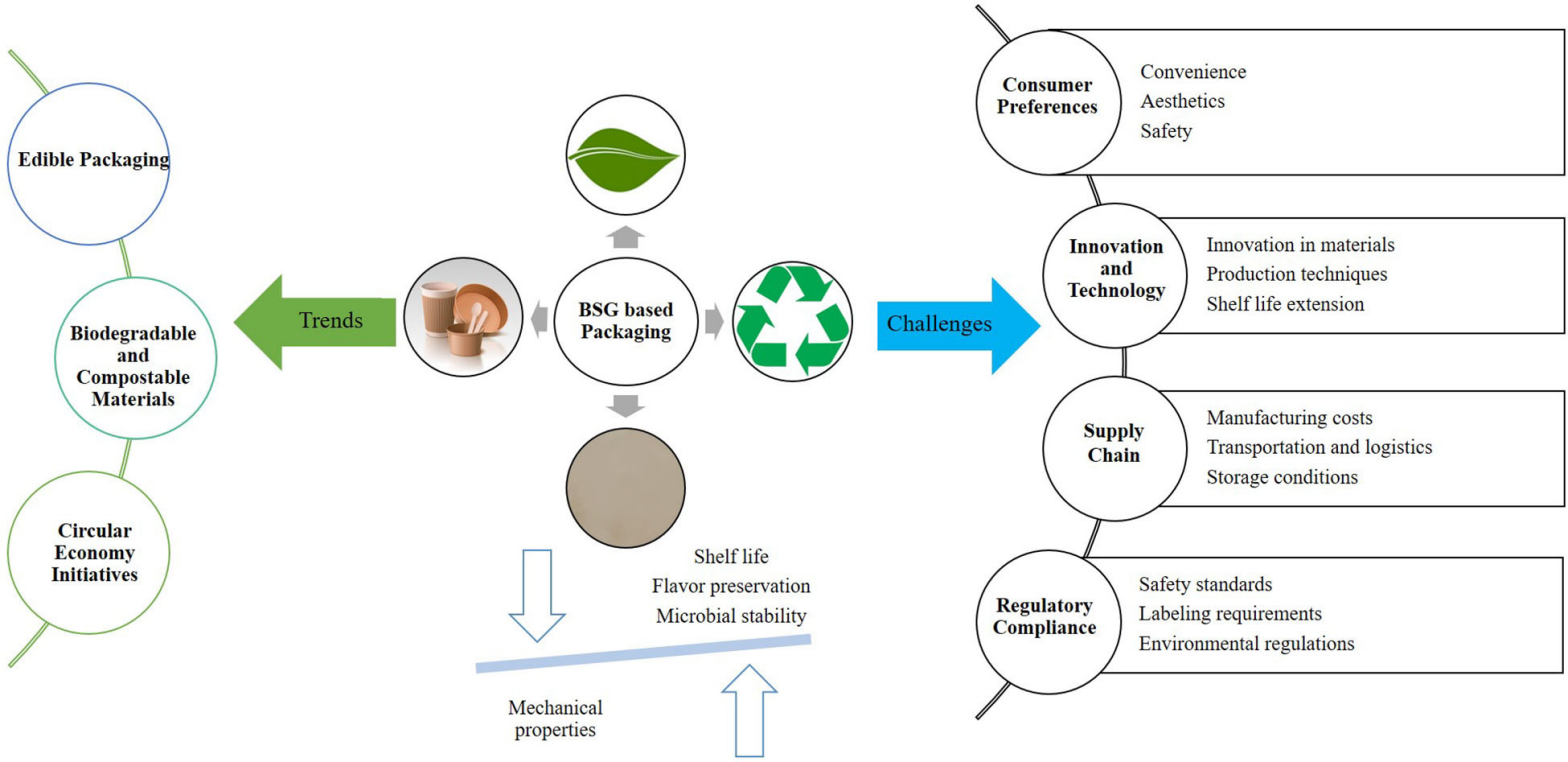
Trends and challenges in BSG‐based packaging material.

BSG‐based lipid, comprising 13% essential oils including tocopherols, tocotrienols, and 5‐*n*‐alkylresorcinols, shows promising properties in the development of food‐packaging materials. Overall, BSG‐based packaging materials exhibit impressive mechanical robustness, hydrophobic characteristics, biodegradability, thermal endurance, UV protection, antibacterial attributes, and antioxidant properties (Figure 2). Incorporating BSG lends the packaging film opaque attributes, underscoring its viability for safeguarding light‐sensitive food items.

## 7 CIRCULAR ECONOMY, SUSTAINABILITY GOAL, AND ENVIRONMENTAL FOOTPRINT

The concept of a circular economy is built upon four key principles: reduce, reuse, recover, and recycle, which aim to optimize economic growth and minimize resource consumption, particularly in terms of materials and energy. The valorization process, which relies on energy‐intensive technologies to transform food byproducts into valuable compounds, may result in little or no GHG savings when compared to alternative products. In sustainability assessments, it was determined that substituting 1 kg of wheat flour with 1 kg of dried BSG leads to a reduction in net greenhouse gas emissions, with a decrease of 0.10 kg CO_2_‐equivalent per kilogram of BSG in food production. This substitution also leads to a reduction in land use (free up 2 m^2^ farm land) (van Deventer et al., 2020). Further studies have demonstrated that transforming BSG into food ingredients not only lowers greenhouse gas emissions but also offers a more sustainable alternative to wheat flour production. Protein substitution from BSG significantly lowers carbon emissions, achieving a reduction of 53.9 kg CO_2_‐eq, whereas replacing acids only achieves a minor decrease of 3.0 kg CO_2_‐eq. Extracting proteins from BSG has been recognized as the process with the greatest potential for progress along the technological evolution scale (Fonseca et al., 2024).

In comparison to biochar production from BSG, which has a GWP of over 1400 million CO_2_ equivalents over a 20‐year period, BSG milling into flour production is expected to have a GWP that is 3.90 times lower (Maqhuzu et al., 2021). Additionally, transitioning from fuel oil to wood biomass chips for thermal energy, combined with utilizing the Norwegian electricity mix, resulted in a significant decrease in emissions—from 115 to 11 kg of CO_2_ equivalent per ton of BSG (Scherhaufer et al., 2020). Other studies have shown that incorporating BSG into aquafeed products can reduce aquaculture environmental footprint by 6%, significantly lowering its overall impact (Iñarra et al., 2022).

The potential of BSG extends beyond food and feed production, as it can also be used to address plastic waste issues. In 2022, the EU generated an estimated 36.1 kg/capita, with only 14.7 kg being recycled (Eurostat, 2022). The cellulose and lignin in BSG have potential applications in materials like biodegradable plastics, composites, and other bio‐based products, reducing reliance on fossil‐fuel‐based materials. Approximately 344,000 t per year of BSG are produced in the EU, which could generate around 6800–13,600 kg of cellulose and 6800–17,000 kg of lignin, reaching a sustainable profit level (de Crane d'Heysselaer et al., 2022). In addition, xylitol is increasingly explored in packaging applications due to its biodegradable, nontoxic, and moisture‐retentive properties. Xylitol production from BSG (101.53 kg/t) is more efficient as compared to polyhydroxybutyrate (10.53 kg/t BSG) (Dávila et al., 2016). The development of BSG protein‐based packaging also presents a promising revenue opportunity, delivering a substantial profit margin. Another potential application of BSG is in its gasification for heat generation in craft breweries, where it has been shown to contribute a net energy output of 28.9 MWh per year. This corresponds to a 22% reduction in the consumption of fossil fuels in the brewing process, leading to significant economic savings (Ortiz et al., 2019).

Despite the growing market for high‐value food ingredients, the amount of BSG utilized in food formulations remains limited. To achieve significant utilization of the BSG side stream, options like using innovative solutions (packaging materials, animal or aquatic feed, pellet production, component extraction) are crucial. However, the lack of studies applying life cycle assessment and life cycle costing to compare alternative BSG management pathways should be addressed in future research, as this would provide valuable insights to inform the decision‐making process.

## 8 CHALLENGES AND FUTURE PROSPECTS

Utilizing BSG for food and packaging applications has few key challenges that require attention and resolution.

### 8.1 Spoilage and safety issue

The high moisture content (75%–85%), along with significant polysaccharide and protein levels, makes BSG highly susceptible to microbial contamination and toxin production. This results in instability, a limited shelf life, and degraded nutritional quality. The sources of variability and the magnitude of microbiome in BSG are influenced by several factors, including raw material, brewing process (techniques, temperatures, and adjuncts used), and storage conditions (temperature, humidity, and duration). Once the sources of variability and the magnitude of the microbiome in BSG are understood, prioritization can be done based on factors such as potential safety risks, impact on product quality, and intended end use.

Effectively managing the microbiome in BSG involves proper storage of wet BSG and processing techniques (drying, heat treatment) to minimize microbial growth. By implementing these strategies, brewers can effectively manage the microbiome in BSG, ensuring its quality, safety, and suitability for various applications in food production. However, specific research on the microbiome BSG is limited; further research in this area could provide valuable insights into the microbial ecology of BSG and its potential applications.

### 8.2 Processing

Drying is the most important process after postproduction of BSG for enhancing the shelf life as well as product safety and quality. However, no standard procedure or consistent guidelines are available for selecting optimal drying parameters in BSG drying. Drying methods include convection drying, drum, infrared, freeze, and oven drying, each of which can impact the nutritional content of BSG differently. Excessive heat during drying can lead to Maillard reactions, which can impact flavor and color. The majority of these studies failed to explicitly quantify how various processing parameters associated with different drying techniques influence nutrient retention in dried BSG. Most of the time, these nutritional losses outweigh changes in product quality that result from utilizing various drying techniques or processing settings by a substantial margin. This poses a challenge for engineers and food technologists to tackle these two interconnected drying problems.

BSG is often generated in large quantities and either needs to be processed immediately or stored properly to prevent spoilage. Convective drying emerges as the most economically viable method, rendering it particularly well‐suited for preserving BSG for diverse applications. Convective drying is favored in the food industry for its cost‐effectiveness, simplicity, versatility, scalability, and efficiency. Its simplicity and compatibility with existing infrastructure make it an attractive option for many facilities, offering easy control over parameters like temperature and airflow. Moreover, integrating non‐invasive methods and sensor techniques into the hot‐air drying process can significantly elevate both food safety and quality control standards while optimizing energy efficiency and overall performance. While other drying methods have their advantages for specific applications, the comprehensive benefits of convective drying make it a popular and enduring choice in the food industry. Overall, convective drying stands out for its comprehensive benefits and enduring popularity in food processing.

Large quantities of BSG are to be processed to prevent spoilage. Storage, drying, milling, and post‐processing steps may include such as baking, heat treatment, fermentation, or enzyme treatment may also be applied to enhance the functional properties of the flour. This cumulative effect of processing on these parameters depends on factors such as processing conditions, equipment used, and the initial quality of the BSG. Proper processing can help retain or enhance the nutritional value of BSG flour while improving its functionality and sensory attributes. Throughout the processing steps, quality control measures should be implemented to ensure consistency and safety of the final product. These measures may regular testing for moisture content, microbial contamination, and nutritional composition. In summary, the overall processing of BSG to flour involves storage, drying, milling, and post‐processing steps, each of which can impact the quality parameters of the final product. Proper processing techniques and quality control measures are essential to ensure the production of high‐quality BSG flour with desirable nutritional, sensory, and functional properties.

### 8.3 Food

The utilization of BSG in food remains limited due to challenges associated with the indigestibility of lignocelluloses and the denaturation state of its protein, which hampers its solubility and industrial application. Most studies suggest incorporating BSG in food products within a 5%–20% range, primarily due to its negative impact on sensory properties, although newer products like chip variants have been developed with up to 40% BSG inclusion. However, substituting BSG often leads to undesirable outcomes such as increased firmness, altered taste, reduced volume expansion, darker coloration, and decreased elasticity.

Storage and drying methods significantly influence flavor and color development, and optimizing these processes can yield high‐quality dried BSG. Furthermore, improving physical properties can be achieved through milling methods, fermentation, extrusion, and modifications using enzyme treatments, dough enhancers, texture modifiers, and dry fractionation to extract protein and fiber components. These techniques enhance particle size distribution, uniformity, functional properties, and nutrient accessibility, thereby improving product quality. Additionally, they enable the development of customized food products with desired nutritional profiles. The fractioned components can be utilized in various food products such as meat analogs, protein bars, dairy alternatives, beverages, sports nutrition products, and baked goods to boost fiber content and enhance digestive health.

### 8.4 Packaging

Laboratory‐scale experiments have demonstrated the potential of BSG in developing innovative packaging materials. However, further research efforts should be directed toward addressing key aspects such as mechanical properties, shelf life, flavor preservation, microbial stability, food‐package interaction, and adherence to food safety standards. Additionally, investigations should prioritize the systematic design of process flows, assessing their technical feasibility and environmental sustainability.

BSG‐derived compounds, like protein, lignin, fiber, and lipid, are pivotal in packaging development. BSG proteins, particularly, offer a cost‐effective alternative for producing biodegradable films with antioxidant properties, suitable for active food packaging. Nevertheless, enhancing the ductility of these films is crucial for practical applications. To improve the mechanical properties of BSG, various treatments such as drying, mercerization, acid hydrolysis, enzymatic treatment, bleaching, and solvent extraction can be employed to eliminate non‐cellulosic compounds, amino acids, and starch. Additionally, improving the composition of the films and refining their processing methods will contribute to better mechanical characteristics. Extraction techniques for BSG should be selected based on target compounds, energy consumption, and production costs. Physical pretreatments like ultrasonication, microwaving, mechanical disruption, and steam explosion are favored for increasing extraction efficiency while minimizing energy consumption, especially given the substantial quantity of brewery byproduct.

The utilization of BSG waste can extend to diverse applications such as paperboard or cardboard, trays, insulation material, mulch and filler, animal feed packaging, and seedling trays. Leveraging these opportunities allows breweries to contribute to the circular economy by repurposing waste materials, thus reducing environmental impact. Collaborations with brewing industries can further propel the advancement of biodegradable packaging, fostering partnerships toward a greener, more sustainable future.

## 9 CONCLUSIONS

The potential of BSG in the food and packaging industry is vast and promising, offering numerous opportunities for innovation and sustainability. Through innovative approaches, BSG can be repurposed to add nutritional value to various food products while simultaneously reducing waste and environmental impact. Its fibrous composition and rich protein and nutrient profile make it a valuable ingredient for enhancing the nutritional content of foods such as bread, snacks, meat analog, yoghurt, and beverages. In addition to its benefits in food, BSG holds great promise in the development of biodegradable packaging, providing a sustainable alternative to conventional materials and contributing to the global effort to reduce plastic pollution. However, further research and development are needed to optimize processing methods, ensure safety standards, and scale up production to meet industry demands. By embracing the potential of BSG and fostering collaboration among brewers, food manufacturers, and packaging companies, we can unlock new opportunities for innovation, sustainability, and economic growth in the food and packaging sectors.

## AUTHOR CONTRIBUTIONS

**Pramod Aradwad**: Conceptualization; methodology; data curation; writing—original draft; formal analysis. **Sharvari Raut**: Conceptualization; methodology; writing—original draft. **Ahmed Abdelfattah**: Writing—review and editing; investigation; visualization. **Cornelia Rauh**: Writing—original draft; writing—review and editing; supervision. **Barbara Sturm**: Conceptualization; writing—original draft; writing—review and editing; supervision; investigation.

## CONFLICT OF INTEREST STATEMENT

The authors declare no conflicts of interest that could have appeared to influence the review work.
